# Postpartum anthropometric and cardiovascular profile following hypertensive disorders of pregnancy: 12‐month follow‐up

**DOI:** 10.1002/uog.70313

**Published:** 2026-09-28

**Authors:** Y. Chen, I. S. Wong, X. Chen, J. Lin, J. Liu, F. Liu, S. L. Lau, Q. Zhang, X. Xu, A. P. W. Lee, L. C. Poon

**Affiliations:** ^1^ Department of Obstetrics and Gynaecology, Prince of Wales Hospital The Chinese University of Hong Kong Hong Kong SAR China; ^2^ Reproductive Center First Teaching Hospital of Tianjin University of Traditional Chinese Medicine, and National Clinical Research Center for Chinese Medicine Acupuncture and Moxibustion Tianjin China; ^3^ Department of Obstetrics and Gynecology, Fujian Maternity and Child Health Hospital Fujian Medical University Teaching Hospital Fujian China; ^4^ Department of Medicine & Therapeutics, Prince of Wales Hospital The Chinese University of Hong Kong Hong Kong SAR China; ^5^ Shenzhen Research Institute The Chinese University of Hong Kong Hong Kong SAR China

**Keywords:** anthropometric, cardiovascular, echocardiography, hypertensive disorders of pregnancy, postpartum, pre‐eclampsia, risk factor

## Abstract

**Objectives:**

To compare anthropometric and cardiovascular parameters at 6–12 weeks postpartum among women classified at first‐trimester screening as high risk for pre‐eclampsia (PE) who subsequently developed hypertensive disorders of pregnancy (HDP), those classified as high risk who did not develop HDP and those classified as low risk for PE, and, among high‐risk women, to evaluate the trajectory of these parameters from 6–12 weeks to 12 months postpartum according to the subsequent development of HDP.

**Methods:**

This was a prospective postpartum longitudinal study conducted at the Prince of Wales Hospital, Hong Kong Special Administrative Region, in which women underwent postpartum assessment between October 2022 and January 2025. Eligible participants were Chinese women in the postpartum period following a singleton pregnancy who had undergone first‐trimester PE screening at 11 + 0 to 13 + 6 weeks' gestation. Women were classified into three groups based on their first‐trimester screening result and subsequent pregnancy outcome: (1) women at high risk (≥ 1:100) for PE who subsequently developed HDP; (2) women at high risk for PE who did not develop HDP; and (3) low‐risk controls with a normotensive pregnancy. Participants were enrolled between 6 and 12 weeks postpartum for anthropometric and cardiovascular assessments, and these findings were compared among the groups. Subsequently, women at high risk for PE were examined longitudinally at 6 months and 12 months postpartum to assess changes in anthropometric and cardiovascular parameters. High‐risk women received lifestyle counseling at each visit. Linear mixed‐effects analysis was performed to compare the longitudinal changes in anthropometric and cardiovascular parameters over the postpartum period.

**Results:**

Overall, 201 women were included in the study, of whom 130 were at high risk for PE and did not develop HDP, 41 were at high risk for PE and developed HDP and 30 were low‐risk controls. At 6–12 weeks postpartum, high‐risk women with HDP exhibited a significantly higher mean arterial pressure, left ventricular (LV) mass index (MI), systemic vascular resistance, myocardial performance index and more impaired global longitudinal strain (GLS), along with lower mitral annular plane systolic excursion, compared with the low‐risk and high‐risk‐without‐HDP groups. Similar findings mostly persisted over the 12‐month postpartum period, even after adjusting for risk factors (all *P* < 0.05). From 6 to 12 weeks to 12 months postpartum, the high‐risk‐without‐HDP group showed a statistically significant declining trend in weight, waist circumference and waist‐to‐hip ratio, along with reduced weight gain from the first trimester to 12 months postpartum, whereas the high‐risk‐with‐HDP group showed no significant change during the same period. The high‐risk‐without‐HDP group exhibited a decreasing trend in LV‐MI and increasing trends in LV ejection fraction, GLS, left atrial emptying fraction and left atrial expansion index from 6–12 weeks to 6 months postpartum. Conversely, in the high‐risk‐with‐HDP group, these improvements only became apparent at 12 months postpartum (all *P* < 0.05).

**Conclusions:**

Women identified as being at high risk for PE at first‐trimester screening, particularly those who develop HDP, exhibit consistently suboptimal anthropometric and cardiovascular trajectories during the first year postpartum. The observed association between maternal risk factors and an unfavorable cardiovascular profile underscores the need to extend anthropometric and cardiovascular screening and implement personalized interventions beyond the standard 6–12‐week postpartum visit in women with HDP. However, the feasibility and practicality of postpartum cardiovascular surveillance are limited by disparities in access to echocardiography, resource allocation for prolonged follow‐up and challenges in improving patient engagement in postpartum care. © 2026 The Author(s). *Ultrasound in Obstetrics & Gynecology* published by John Wiley & Sons Ltd on behalf of International Society of Ultrasound in Obstetrics and Gynecology.

## INTRODUCTION

Hypertensive disorders of pregnancy (HDP) affect up to 10% of pregnancies, with pre‐eclampsia (PE) accounting for 2–5% of these cases^1^. Women with a history of HDP have increased risks of chronic hypertension, metabolic syndrome and cardiovascular disease 3–15 years postpartum^2^, ^3^, ^4^, ^5^, ^6^, ^7^, ^8^, ^9^, ^10^, ^11^, ^12^, ^13^.

Maternal hemodynamic changes from preconception through to the postpartum period represent a continuum of cardiovascular vulnerability, which underscores that cardiovascular maladaptation precedes pregnancy, shapes the clinical expression of HDP and continues to influence physiological recovery well after delivery^14^. Key abnormalities including elevated systemic vascular resistance (SVR), increased left ventricular (LV) mass index (MI) and impaired cardiac function are features of PE. These abnormalities can be detected before pregnancy in women with chronic hypertension or latent cardiovascular dysfunction, indicating that these cardiovascular patterns predate conception. They may also predispose women to HDP and persist for up to 12–18 months postpartum, contributing to the risk of recurrent PE and long‐term CVD^15^, ^16^. Distinct hemodynamic profiles observed in PE‐affected pregnancies, such as a hypodynamic profile in early‐onset PE and hyperdynamic profile in late‐onset PE^17^, may lead to divergent patterns of postpartum LV remodeling and vascular recovery. Impaired venous return and preload reserve may contribute to a high‐resistance, low‐output phenotype in early‐onset PE^18^, and may influence postpartum recovery trajectories.

Despite substantial evidence of cardiac changes following HDP, current guidelines do not recommend echocardiography for risk assessment in this population. The postpartum period, the ‘fourth trimester’, offers a valuable window for identifying women at high risk for future CVD^19^, ^20^. The American Heart Association (AHA) recommends regular postpartum screening for cardiac risk factors in mothers, starting at 6 weeks after delivery, with follow‐up at 6 and 12 months postpartum^21^, ^22^, ^23^. Current studies evaluating cardiac function after HDP are largely limited to the peripartum period or extend into the postpartum period, with assessments typically between 1–3 months and 9–12 months after delivery^21^, ^24^, ^25^, ^26^, and there are limited longitudinal data on cardiovascular profiles in women at high risk for PE throughout the first postpartum year.

This study aimed to: (1) compare anthropometric and cardiovascular parameters at 6–12 weeks postpartum among women at low risk for PE, women at high risk for PE who subsequently developed HDP and women at high risk for PE who did not develop HDP, as determined by first‐trimester screening; and (2) among women at high risk for PE, evaluate the trajectory of these parameters from 6–12 weeks to 12 months postpartum according to subsequent development of HDP.

## METHODS

### Study design

This was a prospective postpartum longitudinal study conducted at the Prince of Wales Hospital, Hong Kong Special Administrative Region, China, in which women underwent postpartum assessment between October 2022 and January 2025. Eligible participants were Chinese women in the postpartum period following a singleton pregnancy who had undergone first‐trimester PE screening at 11 + 0 to 13 + 6 weeks' gestation.

Women identified as being at high risk for PE (risk ≥ 1:100) on first‐trimester screening had received aspirin prophylaxis as part of our standard protocol, initiated before 16 weeks at a dose of 160 mg per day and continued until 36 weeks, delivery or diagnosis of PE^27^.

HDP was defined, according to the International Society for the Study of Hypertension in Pregnancy (ISSHP) guideline^1^, as a diagnosis of PE or gestational hypertension, with diagnostic criteria as defined in the ISSHP guideline. PE was further subclassified based on gestational age at delivery, with early‐onset PE defined as delivery at < 34 + 0 weeks and late‐onset PE defined as delivery at ≥ 34 + 0 weeks^28^.

Based on their first‐trimester PE screening result and subsequent pregnancy outcome, eligible women were invited via phone call to attend a postpartum assessment between 6 and 12 weeks after delivery and were classified into three groups: (1) women at high risk for PE who developed HDP; (2) women at high risk for PE who did not develop HDP; and (3) women at low risk for PE who had a normotensive pregnancy. Postpartum anthropometric and cardiovascular parameters were subsequently compared across these groups to evaluate the associations of antenatal PE risk status and the development of HDP with maternal recovery after pregnancy.

The low‐risk group served as a baseline reference group for comparisons of cardiovascular parameters at 6–12 weeks postpartum. Of note, the low‐risk group was assessed only at 6–12 weeks postpartum and did not undergo longitudinal follow‐up. Subsequently, women in the two high‐risk groups underwent additional longitudinal assessments at 6 months and 12 months postpartum. Participants were excluded if they had a new pregnancy, pre‐existing chronic hypertension or maternal heart disease, or were unable to provide written informed consent, had learning difficulties or were unable to understand spoken and written Chinese or English.

Maternal demographic and clinical characteristics, including age, parity, smoking status, pre‐existing chronic diseases and obstetric history, were collected. Pregnancy outcomes, including gestational age at delivery and perinatal outcomes, were obtained from hospital maternity records. All eligible women provided written informed consent before participation. Approval for this study was obtained from the Joint Chinese University of Hong Kong‐New Territories East Cluster Clinical Research Ethics Committee (CREC Ref. No. 2022.340).

### Anthropometric assessment

Maternal weight and height were measured, and body mass index (BMI) was calculated. Waist circumference was measured at the midpoint between the lowest ribs and the iliac crest. Hip circumference was measured horizontally at the level of the most prominent lateral extension of the hips. The waist‐to‐hip ratio was calculated by dividing waist circumference (in cm) by hip circumference (in cm).

### Cardiovascular assessment

Blood pressure was measured using validated automated devices (BP3AQ1; Microlife, Taipei, Taiwan)^29^. Mean arterial pressure (MAP) was calculated using the formula: (2 × diastolic blood pressure + systolic blood pressure)/3^30^. Transthoracic echocardiography was performed with the woman positioned in the left lateral decubitus position with her left arm behind her head. Two‐dimensional and color‐Doppler transthoracic echocardiography was performed according to international guidelines^31^, ^32^. A PA1‐5A phased‐array transducer and a V8 ultrasound system (Samsung Medison, Seoul, South Korea) were used for echocardiographic examination. Echocardiography was performed by a sonographer (Y.C.) trained and certified in the acquisition and analysis of echocardiograms. The sonographer was blinded to the medical history of the patient when obtaining and analyzing echocardiographic data. Data were acquired from the standard views, including the parasternal long‐axis, apical four‐chamber, two‐chamber, three‐chamber and five‐chamber views. For each acquisition, a minimum of three consecutive cardiac cycles were obtained for the measurement of conventional and tissue‐Doppler parameters. Echocardiographic measurements, including cardiac hemodynamics, geometry and function, were performed following the standardized protocol in Appendices S1 and S2, ^31^, ^33^, ^34^. The HeartAssist™ program (Samsung Medison) was used to automatically measure the velocity time integral, LV‐MI, relative wall thickness (RWT) and septal and lateral mitral annular e′ velocity. At each visit, women in the high‐risk groups received cardiovascular risk counseling and advice on lifestyle modifications to reduce cardiovascular risk, including sustained breastfeeding, regular physical activity and adherence to a healthy diet.

### Sample‐size calculation

Given the lack of existing data on maternal cardiovascular parameters from 6–12 weeks to 6 months postpartum among women identified as being at high risk for PE who subsequently did or did not develop HDP, the sample size was calculated using Glimmpse version 3.0.0 (http://glimmpse.samplesizeshop.org/). The calculation was based on the effect size observed for changes in SVR between women at high risk for PE and low‐risk controls from the third trimester of pregnancy (SVR: 852 ± 190 dyn × s/cm^5^ *vs* 1129 ± 318 dyn × s/cm^5^) to 4 months postpartum (SVR: 1134 ± 257 dyn × s/cm^5^ *vs* 1062 ± 291 dyn × s/cm^5^)^35^. While this comparison does not exactly align with the primary analysis in this study between women classed as high risk for PE with and those without a subsequent diagnosis of HDP, it provides a close estimate of effect size for the sample‐size calculation, given the paucity of published longitudinal data within the high‐risk population. At a power of 80%, a Type‐1 error rate of 0.05 and an anticipated 20% rate of missing data, 210 cases in total were needed. Assuming the incidence rate of HDP among high‐risk women to be 20%, the target recruitment was 180 high‐risk women and 30 low‐risk women, with at least 36 women with HDP.

### Statistical analysis

All baseline characteristics, such as maternal demographic characteristics, medical history and obstetric history, were compared descriptively among the three study groups (high‐risk women who developed HDP, high‐risk women who did not develop HDP and low‐risk women). Continuous data are presented as median (interquartile range) for non‐normally distributed data or mean ± SD for normally distributed data, while categorical data are expressed as *n* (%). Categorical variables were compared using the chi‐square test or Fisher's exact test, as appropriate. Normality of the data was tested by the Kolmogorov–Smirnov test. Between‐group comparisons were assessed using Student's *t*‐test and one‐way ANOVA for normally distributed data, and the Mann–Whitney *U*‐test and Kruskal–Wallis test for non‐normally distributed data. To examine potential attrition bias, baseline characteristics were compared between women included in the final analysis and those excluded from the study, using Student's *t*‐test, the Mann–Whitney *U*‐test, the chi‐square test or Fisher's exact test, as appropriate.

To compare changes in anthropometric and cardiovascular parameters from 6–12 weeks to 12 months postpartum between the two high‐risk groups, linear mixed‐effects model analysis, including fixed and random effects, was performed. This model allows for flexibility to account simultaneously for repeated measurements from the same participants at different visits (within subjects) and to address the differences between participants (between subjects). The fixed‐effect components included clinical visits (three study timepoints), study group, age, height, weight, smoking, family history of CVD, parity, history of previous HDP, aspirin prophylaxis during pregnancy, gestational diabetes mellitus, any breastfeeding and the interaction between clinical visit and study group for each variable. The random‐effect components consisted of the intercept (participant identity) and the slope (clinical visits). The best‐fit models (comparing the base model without random effects, models with a random slope only for clinical visits, random intercept or both random intercept and slope) were selected based on the lowest Akaike information criterion. The values of the estimated marginal mean (95% CI) along with effect sizes (Cohen's *d*) of each cardiovascular parameter at each study timepoint are presented in the supplementary material. To account for multiple comparisons across time points and groups, Holm–Bonferroni correction was employed, with a family‐wise significance level of 0.05, by sequentially adjusting the per‐comparison alpha threshold based on the rank order of *P*‐values^36^. *P* < 0.05 was considered statistically significant for unadjusted pairwise comparisons. Data analysis was performed using R version 4.1.3 (The R Foundation for Statistical Computing, Vienna, Austria)^37^.

## RESULTS

### Participant characteristics

In total, 267 women were recruited for anthropometric and cardiovascular assessment at 6–12 weeks postpartum. Of these, 18 (6.74%) cases lost to follow‐up at 6 months postpartum, two (0.75%) cases with arrhythmia, five (1.87%) cases with poor image quality, seven (2.62%) cases with pre‐existing chronic hypertension and 34 (12.73%) cases lost to follow‐up at 12 months postpartum were excluded. The remaining 201 (75.28%) women were included in the final analysis, of whom 130 were classified during first‐trimester screening as being at high risk for PE and did not develop HDP (high‐risk‐without‐HDP group), 41 were classified as being at high risk for PE and developed HDP (high‐risk‐with‐HDP group) and 30 were classified as low risk and had normotensive pregnancies (low‐risk group). Baseline characteristics were comparable between women included in the final analysis and those excluded from the study (Table S1). Of the nine high‐risk women who had developed chronic hypertension by 12 months postpartum, two were on antihypertensive medication.

The demographic characteristics and pregnancy outcomes of the three study groups are summarized in Table 1. Compared with the low‐risk group, both high‐risk groups had a higher prevalence of previous HDP, higher MAP raw values and multiples of the median, and a higher predicted risk for PE at first‐trimester PE screening. All high‐risk women who used aspirin demonstrated good or moderate adherence during pregnancy. In the high‐risk‐with‐HDP group, 19 women developed gestational hypertension and 22 developed PE, including 10 cases with early‐onset PE and 12 cases with late‐onset PE. The high‐risk‐with‐HDP group had lower median gestational age at delivery, birth weight and 1‐ and 5‐min Apgar scores compared with the other two groups.

**Table 1:** Maternal characteristics of postpartum study population according to first‐trimester pre‐eclampsia (PE) risk status and subsequent development of hypertensive disorders of pregnancy (HDP)

Variable	Low risk without HDP (*n* = 30)	High risk without HDP (*n* = 130)	High risk with HDP (*n* = 41)	*P*
Maternal age at 6–12 weeks postpartum (years)	33.40 ± 4.59	36.30 ± 4.17	34.94 ± 5.11	0.0037*
Maternal height at 6–12 weeks postpartum (m)	1.59 ± 0.05	1.58 ± 0.05	1.58 ± 0.06	0.70
Obstetric history				
Parity	1 (1–1)	1 (1–1)	1 (1–2)	0.09
Type‐1 or Type‐2 diabetes mellitus	0 (0)	1 (0.77)	1 (2.44)	0.54
Family history of CVD	8 (26.67)	64 (49.23)	22 (53.66)	0.51
History of previous HDP	0 (0)	8 (6.15)	8 (19.51)	0.0049*, †
First‐trimester weight (kg)	59.99 ± 10.27	59.20 ± 9.87	63.00 ± 10.23	0.11
First‐trimester MAP (mmHg)	80.77 ± 6.79	86.42 ± 7.68	92.21 ± 9.89	< 0.001*, †, ‡
First‐trimester MAP MoM	0.99 (0.96–1.08)	1.08 (1.02–1.15)	1.13 (1.07–1.21)	< 0.001*, †
First‐trimester UtA‐PI MoM	1.06 ± 0.25	1.19 ± 0.27	1.11 ± 0.26	0.02*
First‐trimester PlGF MoM	1.12 (0.88–1.26)	0.57 (0.41–0.77)	0.64 (0.49–0.78)	< 0.001*
Predicted risk of PE at first‐trimester screening (1 in *x*)	370.00 (229.25–922.25)	47.50 (28.75–70.50)	32.00 (18.00–59.00)	< 0.001*, †
Aspirin prophylaxis	NA	124 (95.38)	39 (95.12)	NaN
GA at initiation of aspirin prophylaxis (weeks)	NA	13.43 (13.14–14.00)	13.57 (13.21–14.00)	0.31
Aspirin adherence (%)	NA	99.4 (98.0–100.0)	99.2 (95.6–100.0)	0.31
Good adherence		121/124 (97.58)	35/39 (89.74)	0.072
Moderate adherence		3/124 (2.42)	4/39 (10.26)	
GDM	0 (0)	21 (16.15)	4 (9.76)	0.046
Subtype of HDP				
Gestational hypertension	0 (0)	0 (0)	19 (46.34)	< 0.01†, ‡
PE	0 (0)	0 (0)	22 (53.66)	< 0.01†, ‡
Early‐onset PE (< 34 weeks)	0 (0)	0 (0)	10 (24.39)	< 0.01†, ‡
Late‐onset PE (≥ 34 weeks)	0 (0)	0 (0)	12 (29.27)	< 0.01†, ‡
GA at delivery (weeks)	39.21 (38.46–39.82)	38.29 (37.89–39.14)	37.14 (35.29–37.86)	< 0.0001*, †, ‡
Birth weight (g)	3102.50 (2891.25–3330.00)	2890.00 (2650.00–3160.00)	2625.00 (2160.00–2910.00)	0.0001†, ‡
1‐min Apgar score	9 (9–9)	9 (8–9)	8 (7–9)	0.0002†, ‡
5‐min Apgar score	10 (10–10)	10 (9.5–10)	10 (8–10)	0.0004†, ‡
Breastfeeding				
Breastfeeding (any)	23 (76.67)	114 (87.69)	36 (87.80)	0.297
Breastfeeding ≥ 6 weeks	17 (56.67)	92 (70.77)	27 (65.85)	0.318
Exclusive breastfeeding ≥ 6 weeks	2 (6.67)	13 (10.00)	5 (12.20)	0.744
Breastfeeding ≥ 6 months	NA	57 (43.85)	21 (51.22)	0.473
Exclusive breastfeeding ≥ 6 months	NA	7 (5.38)	4 (9.76)	0.464

Data are given as mean ± SD, median (interquartile range), *n* (%) or *n*/*N* (%). *P*‐values for overall comparisons between groups at each time point are presented. *†‡Significant differences on pairwise comparisons:

* significant difference between women at low risk for PE and women at high risk for PE without HDP;

† significant difference between women at low risk for PE and women at high risk for PE with HDP;

‡ significant difference between women at high risk for PE with HDP and women at high risk for PE without HDP. CVD, cardiovascular disease; GA, gestational age; GDM, gestational diabetes mellitus; MAP, mean arterial pressure; MoM, multiples of the median; NA, not applicable; NaN, not a number; PlGF, placental growth factor; UtA‐PI, uterine artery pulsatility index.

There was no significant difference in the overall breastfeeding rate or the rate of exclusive breastfeeding ≥ 6 weeks among the three study groups. Similar findings for the overall breastfeeding and exclusive breastfeeding rates ≥ 6 months postpartum were observed between the two high‐risk groups (Table 1).

### Anthropometric parameters

At 6–12 weeks postpartum, there were no significant differences in median weight and BMI among the three study groups (Tables 2, S2 and Figure 1a); both high‐risk groups exhibited a higher waist‐to‐hip ratio from the first trimester to 6–12 weeks postpartum compared with the low‐risk group (Table 2, Figure 1b). All anthropometric parameters were comparable between the two high‐risk groups at 6–12 weeks postpartum, even after multivariable analysis (Table S3).

**Table 2:** Comparison of crude (unadjusted) values of anthropometric and cardiovascular parameters at 6–12 weeks postpartum, according to first‐trimester pre‐eclampsia (PE) risk status and subsequent development of hypertensive disorders of pregnancy (HDP), and between high‐risk women with and those without HDP

	6–12 weeks postpartum				6 months postpartum				12 months postpartum			
Variable	Low risk without HDP	High risk without HDP	High risk with HDP	*P*	High risk without HDP	High risk with HDP	Cohen's *d* (95% CI)	*P*	High risk without HDP	High risk with HDP	Cohen's *d* (95% CI)	*P*
Anthropometrics												
Weight (kg)	64.33 (58.28–68.39)	60.65 (55.52–66.90)	62.90 (58.50–66.90)	0.236	60.90 (54.00–67.40)	62.90 (58.50–70.90)	0.37 (0.02–0.73)	0.084	60.65 (52.45–66.6)	62.50 (58.4–70.1)	0.47 (0.10–0.84)	0.013
Weight gain from first trimester to postpartum period (kg)	4.05 (2.28–5.50)	2.55 (0.43–5.00)	1.60 (–0.40 to 2.60)	0.029†	2.40 (–0.70 to 4.60)	2.20 (–0.20 to 3.70)	–0.01 (–0.22 to 0.19)	0.90	1.50 (–1.20 to 3.80)	2.30 (–1.10 to 4.80)	0.14 (–0.08 to 0.36)	0.20
BMI (kg/m^2^)	24.90 (22.49–26.50)	24.40 (22.18–26.32)	24.76 (23.17–27.68)	0.41	24.14 (21.76–26.75)	25.02 (22.75–28.12)	0.39 (0.04–0.75)	0.08	24.12 (21.10–26.53)	25.31 (23.01–29.42)	0.47 (0.10–0.84)	0.052
Waist circ (cm)	84.08 ± 7.54	86.78 ± 7.73	89.53 ± 8.97	0.019†	85.43 ± 9.10	90.29 ± 8.69	0.54 (0.18–0.90)	0.003	82.96 ± 9.97	88.66 ± 9.99	0.57 (0.21–0.93)	0.005
Hip circ (cm)	99.23 ± 7.32	98.56 ± 7.15	100.12 ± 7.85	0.486	97.77 ± 7.65	100.03 ± 8.78	0.29 (–0.07 to 0.64)	0.113	96.72 ± 7.52	98.86 ± 9.42	0.27 (–0.09 to 0.62)	0.142
Waist‐to‐hip ratio	0.839 (0.814–0.883)	0.883 (0.852–0.913)	0.889 (0.858–0.929)	0.001 *, †	0.878 (0.839–0.904)	0.905 (0.867–0.938)	0.53 (0.17–0.88)	0.003	0.854 (0.815–0.897)	0.899 (0.844–0.929)	0.68 (0.31–1.04)	0.001
Hemodynamics												
MAP (mmHg)	81.25 (77.46–87.73)	82.75 (79.08–88.75)	96.08 (84.42–102.25)	< 0.001†, ‡	83.17 (78.58–89.00)	96.42 (86.75–100.67)	0.98 (0.62–1.35)	< 0.001	83.58 (78.44–89.27)	92.12 (87.27–101.12)	0.93 (0.56–1.30)	< 0.001
SV (mL)	72.58 ± 9.25	62.76 ± 10.56	60.19 ± 8.51	< 0.001*, †	62.45 ± 11.34	61.49 ± 11.88	–0.08 (–0.44 to 0.27)	0.643	61.88 ± 9.95	61.20 ± 10.66	–0.07 (–0.42 to 0.29)	0.713
CO (L/min)	4.45 (4.20–4.89)	4.09 (3.70–4.62)	4.23 (3.76–4.58)	0.012*	4.19 (3.70–4.80)	4.42 (3.93–4.66)	0.03 (–0.32 to 0.38)	0.554	4.20 (3.60–4.71)	4.39 (3.96–4.85)	0.12 (–0.24 to 0.47)	0.31
Heart rate (bpm)	63.00 (59.00–67.75)	66.00 (61.00–74.00)	68.00 (63.00–74.25)	0.018†	69.50 (62.00–75.00)	70.00 (65.00–78.00)	0.19 (–0.16 to 0.54)	0.295	69.00 (62.00–73.00)	72.00 (66.00–77.00)	0.29 (–0.06 to 0.65)	0.063
SVR (dyn × s × cm^−^^5^)	1415.03 (1367.38–1559.01)	1662.98 (1433.42–1819.82)	1782.33 (1541.56–2023.44)	< 0.001*, †, ‡	1563.65 (1360.35–1878.55)	1743.55 (1489.33–2027.79)	0.44 (0.08–0.80)	0.041	1592.97 (1451.62–1876.75)	1678.06 (1500.99–2041.86)	0.32 (–0.05 to 0.68)	0.212
SVRI (dyn × s × cm^−5^ × m^2^)	885.12 (847.30–922.95)	1031.80 (992.35–1071.24)	1104.25 (1031.00–1177.50)	< 0.001*, †, ‡	1011.78 (969.36–1054.20)	1083.48 (998.23–1168.73)	0.28 (–0.07 to 0.64)	0.145	1045.27 (998.50–1092.04)	1080.68 (982.48–1178.87)	0.13 (–0.24 to 0.50)	0.526
Geometry												
LV‐MI (g/m^2^)	61.09 ± 8.88	66.70 ± 10.00	72.33 ± 9.70	< 0.001*, †, ‡	63.15 ± 11.11	68.57 ± 12.65	0.47 (0.12–0.83)	0.009	62.30 ± 10.50	68.04 ± 11.44	0.53 (0.18–0.89)	0.003
RWT	0.28 (0.25–0.31)	0.31 (0.28–0.35)	0.34 (0.30–0.37)	< 0.001*, †, ‡	0.31 (0.27–0.35)	0.32 (0.29–0.37)	0.32 (–0.04 to 0.67)	0.096	0.30 (0.26–0.34)	0.34 (0.27–0.37)	0.55 (0.20–0.91)	0.004
LV‐EDV (mL)	67.92 (61.78–70.24)	70.17 (60.99–79.49)	74.59 (68.31–83.70)	0.012†	67.00 (59.73–75.76)	71.65 (66.30–82.11)	0.56 (0.19–0.92)	0.003	66.16 (59.26–77.52)	71.88 (62.78–79.16)	0.29 (–0.06 to 0.65)	0.073
LV‐ESV (mL)	26.81 (19.86–28.26)	28.04 (23.51–32.61)	30.06 (27.23–34.84)	0.005†	25.49 (21.39–29.21)	29.08 (26.19–33.22)	0.68 (0.31–1.05)	< 0.001	25.04 (21.19–29.87)	27.41 (23.48–34.14)	0.44 (0.08–0.79)	0.037
Systolic function												
LV‐EF	0.643 ± 0.038	0.600 ± 0.043	0.576 ± 0.040	< 0.001*, †, ‡	0.613 ± 0.045	0.591 ± 0.057	–0.46 (–0.82 to –0.10)	0.013	0.622 ± 0.046	0.600 ± 0.055	–0.46 (–0.81 to –0.10)	0.013
MPI	0.332 ± 0.04	0.360 ± 0.05	0.389 ± 0.06	0.001†, ‡	0.35 ± 0.05	0.380 ± 0.05	0.57 (0.21–0.93)	0.002	0.340 ± 0.05	0.370 ± 0.07	0.53 (0.17– 0.89)	0.004
MAPSE (cm)	1.53 ± 0.15	1.57 ± 0.18	1.43 ± 0.20	< 0.001*, †, ‡	1.58 ± 0.18	1.47 ± 0.20	–0.61 (–0.97 to –0.25)	0.001	1.56 ± 0.16	1.50 ± 0.18	–0.36 (–0.73 to 0.00)	0.053
GLS	–0.268 (–0.276 to –0.258)	–0.255 (–0.272 to –0.242)	–0.244 (–0.251 to –0.231)	< 0.001*, †, ‡	–0.266 (–0.277 to –0.252)	–0.249 (–0.262 to –0.235)	0.79 (0.42–1.17)	< 0.0001	–0.267 (–0.275 to –0.254)	–0.258 (–0.264 to –0.244)	0.49 (0.12–0.86)	0.01
Diastolic function												
MV E/A ratio	1.37 (1.10–1.85)	1.29 (1.06–1.53)	1.12 (0.97–1.31)	0.007†	1.31 (1.11–1.55)	1.29 (1.02–1.47)	–0.33 (–0.69 to 0.03)	0.159	1.33 (1.20–1.64)	1.21 (1.02–1.46)	–0.41 (–0.77 to –0.05)	0.037
MV septal e′ velocity (cm/s)	10.47 ± 1.70	10.38 ± 1.91	9.69 ± 2.03	0.113	10.73 ± 2.07	10.25 ± 1.89	–0.23 (–0.59 to 0.12)	0.194	10.85 ± 1.82	10.12 ± 2.12	–0.40 (–0.75 to –0.04)	0.08
MV lateral e′ velocity (cm/s)	13.51 ± 2.11	13.44 ± 2.36	12.36 ± 2.66	0.041‡	13.58 ± 2.22	12.92 ± 2.61	–0.28 (–0.64 to 0.07)	0.116	13.30 ± 2.23	12.86 ± 2.32	–0.20 (–0.55 to 0.16)	0.278
E/e′	5.89 (5.11–6.49)	6.00 (5.27–6.95)	6.15 (5.59–6.81)	0.569	6.24 (5.59–6.93)	6.37 (5.89–7.09)	0.22 (–0.14 to 0.57)	0.262	6.19 (5.46–7.22)	6.53 (5.75–7.61)	0.22 (–0.13 to 0.57)	0.313
LA‐VImax (mL/m^2^)	27.28 (22.63–29.04)	27.10 (22.72–31.06)	27.50 (24.82–30.75)	0.541	25.28 (23.15–29.12)	26.83 (23.79–29.74)	0.18 (–0.18 to 0.54)	0.346	27.30 (24.06–31.57)	27.73 (24.67– 30.82)	0.13 (–0.23 to 0.50)	0.91
LA‐VImin (mL/m^2^)	11.91 (10.09–12.27)	15.24 (12.94–17.35)	16.61 (14.06–19.63)	< 0.001*, †	14.04 (12.50–16.11)	15.64 (13.43–17.93)	0.38 (0.03–0.74)	0.055	13.87 (11.84–16.82)	16.30 (14.00–17.75)	0.51 (0.15–0.88)	0.012
LA emptying fraction	0.550 ± 0.053	0.426 ± 0.053	0.395 ± 0.070	< 0.001*, †	0.441 ± 0.051	0.415 ± 0.084	–0.43 (–0.79 to –0.07)	0.019	0.486 ± 0.059	0.436 ± 0.065	–0.84 (–1.21 to –0.47)	< 0.001
LA‐EI	1.31 (1.19–1.36)	0.74 (0.64–0.89)	0.64 (0.54–0.77)	< 0.001*, †, ‡	0.80 (0.71–0.93)	0.73 (0.55–0.93)	–0.28 (–0.64 to 0.08)	0.04	0.93 (0.82–1.15)	0.78 (0.63–0.93)	–0.73 (–1.10 to –0.36)	< 0.001

Data are given as median (interquartile range) or mean ± SD, unless stated otherwise.

*†‡Significant differences on pairwise comparisons:

* significant difference between women at low risk for PE and women at high risk of PE without HDP;

† significant difference between women at low risk of PE and women at high risk of PE with HDP;

‡ significant difference between women at high risk of PE without HDP and women at high risk of PE with HDP. BMI, body mass index; circ, circumference; CO, cardiac output; E/A ratio, ratio of atrioventricular peak flow velocities at early diastole and atrial contraction; GLS, global longitudinal strain; LA, left atrial; LA‐EI, left atrial expansion index; LA‐VImax, maximum left atrial volume index; LA‐VImin, minimum left atrial volume index; LV‐EDV, left ventricular end‐diastolic volume; LV‐EF, left ventricular ejection fraction; LV‐ESV, left ventricular end‐systolic volume; LV‐MI, left ventricular mass index; MAP, mean arterial pressure; MAPSE, mitral annular plane systolic excursion; MPI, myocardial performance index; MV, mitral valve; RWT, relative wall thickness; SV, stroke volume; SVR, systemic vascular resistance; SVRI, systemic vascular resistance index.

**Figure 1:**
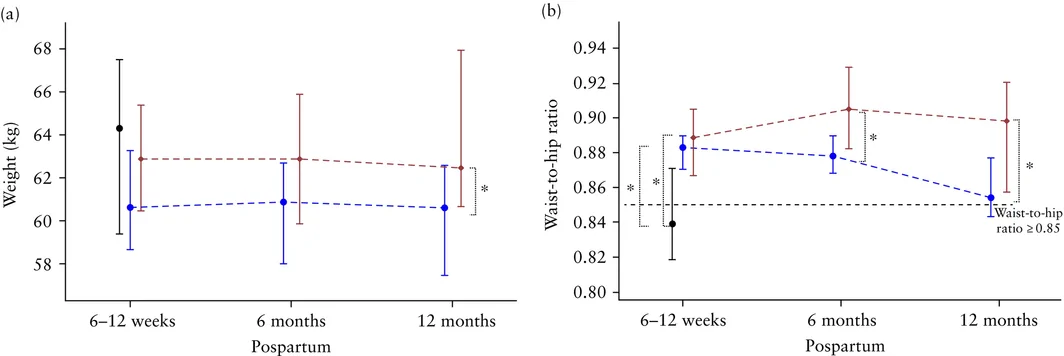
Trajectories of unadjusted median values (with 95% CI) for weight (a) and waist‐to‐hip ratio (b) among women classified as being at low risk for pre‐eclampsia (PE) (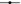), those at high risk for PE who did not develop hypertensive disorders of pregnancy (HDP) (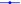) and those at high risk for PE who developed HDP (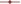), at 6–12 weeks, 6 months and 12 months postpartum. 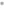
Statistically significant difference between groups.

However, at 6 months postpartum, a clear divergence of anthropometric profiles between the two high‐risk groups was apparent: the high‐risk‐with‐HDP group exhibited a significantly greater waist circumference and higher waist‐to‐hip ratio compared with the high‐risk‐without‐HDP group. These differences persisted to 12 months postpartum (Figure 1b, Table 2). Additionally, at 12 months postpartum, the mean weight of women in the high‐risk‐with‐HDP group was significantly greater than in the high‐risk‐without‐HDP group (Figure 1a). Multivariable analysis confirmed that the high‐risk‐with‐HDP group demonstrated significantly higher BMI and waist‐to‐hip ratio at both 6 and 12 months postpartum compared with the high‐risk‐without‐HDP group (Table S3). Higher parity was associated with greater measurements of weight and BMI (*P* < 0.05, data not shown). Among high‐risk women, those who breastfed had a lower waist‐to‐hip ratio, compared to those who did not (*P* < 0.01, data not shown).

### Hemodynamics and geometry

Regarding cardiac hemodynamic parameters at 6–12 weeks postpartum, univariable analysis showed that the high‐risk‐with‐HDP group had significantly higher MAP than did the other two groups (Tables 2, S2, Figure 2a). The high‐risk‐with‐HDP group also had the highest systemic vascular resistance (SVR) and SVR index (Table 2, Figure 2b), as well as the greatest LV‐MI and RWT, followed by the high‐risk‐without‐HDP group and then the low‐risk group (Figure 2c, Table 2). Similarly, at 6 months postpartum, the high‐risk‐with‐HDP group demonstrated significantly higher MAP and SVR (Figure 2a,b), along with signs of cardiac remodeling, such as increased LV‐MI (Figure 2c), left ventricular end‐diastolic volume (LV‐EDV) and LV end‐systolic volume (LV‐ESV) compared with the high‐risk‐without‐HDP group (Table 2). These findings persisted until 12 months postpartum for MAP and LV‐ESV, while no significant difference between the two groups was observed in SVR or LV‐EDV at that time (Table 2). Similarly, after multivariable analysis, among high‐risk women, those with HDP demonstrated significantly higher MAP, SVR and LV‐MI throughout the 12‐month postpartum period (Tables S3 and S4). The significant differences in LV‐ESV between the high‐risk groups persisted up to 6 months postpartum (Table S4). Furthermore, increased weight and age, a family history of CVD and a history of previous HDP were associated with suboptimal cardiac hemodynamic and geometrical changes, such as higher MAP and LV‐MI, among high‐risk women (all *P* < 0.05, data not shown).

**Figure 2:**
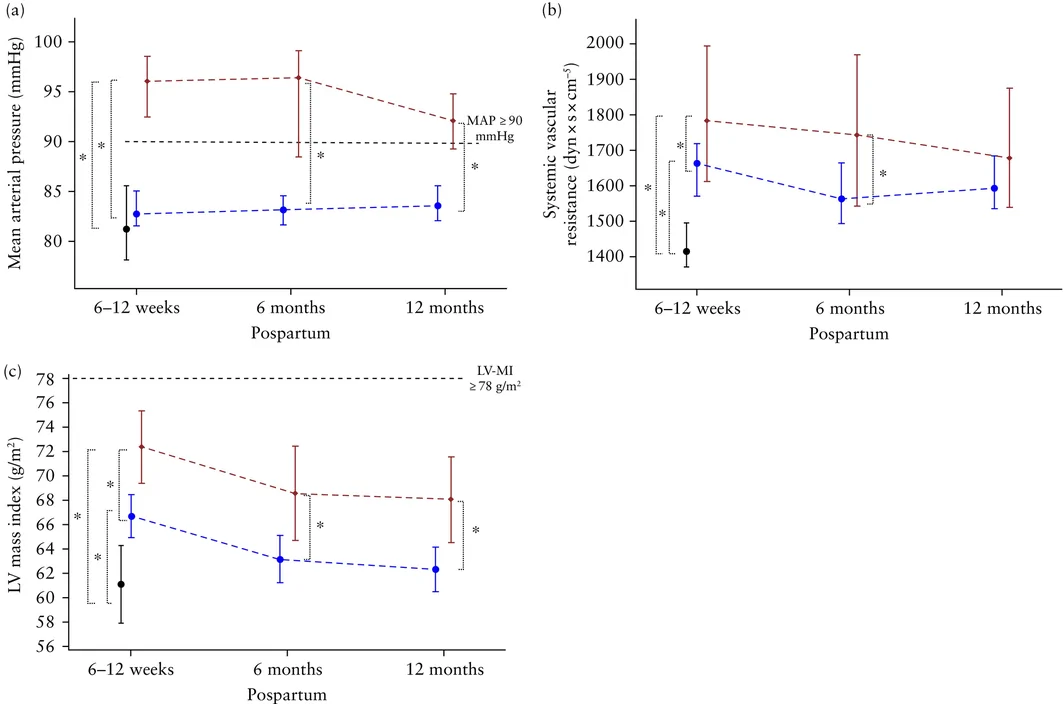
Trajectories of unadjusted median values (with 95% CI) for mean arterial pressure (a) and systemic vascular resistance (b), and unadjusted mean values (with 95% CI) for left ventricular mass index (c), among women classified as being at low risk for pre‐eclampsia (PE) (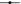), those at high risk for PE who did not develop hypertensive disorders of pregnancy (HDP) (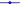) and those at high risk for PE who developed HDP (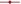), at 6–12 weeks, 6 months and 12 months postpartum. 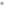
Statistically significant difference between groups.

### Systolic function

At 6–12 weeks postpartum, the high‐risk‐with‐HDP group demonstrated the most impaired systolic profile, indicated by the lowest LV ejection fraction (EF) (Figure 3a) as well as impaired global longitudinal strain (GLS) (Figure 3b), followed by the high‐risk‐without‐HDP group and then the low‐risk group (Table 2). Similarly, this pattern of impaired function in the high‐risk‐with‐HDP group persisted at 6 and 12 months postpartum, as evidenced by most of the systolic functional parameters, in comparison with the high‐risk‐without‐HDP group (Table 2).

**Figure 3:**
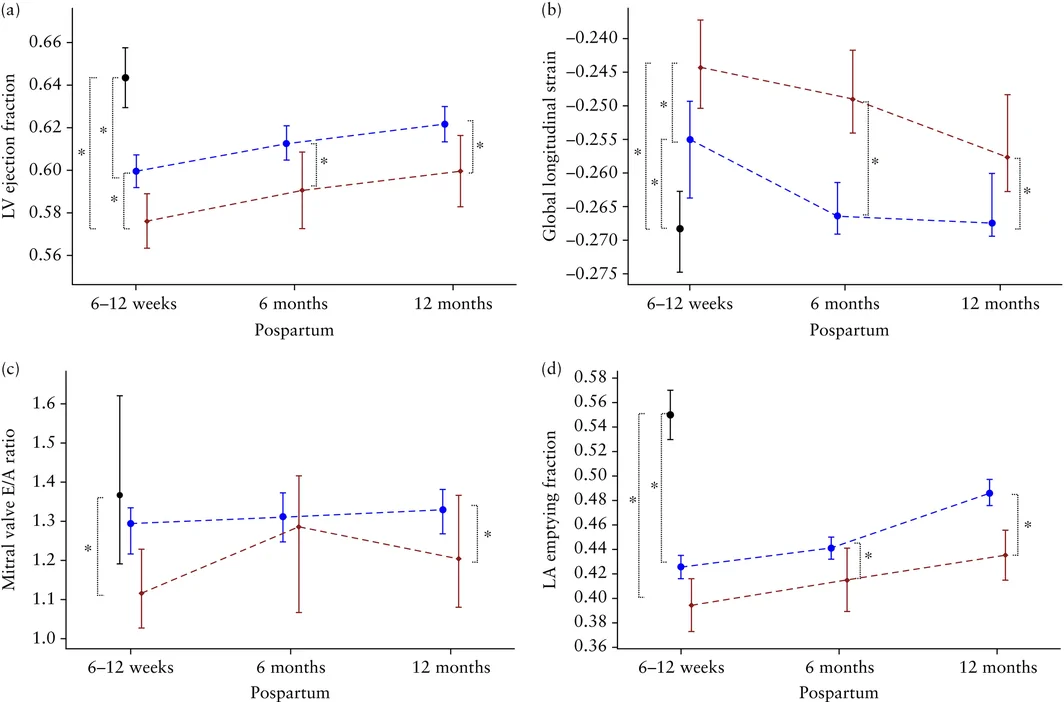
Trajectories of unadjusted mean (a,d) and median (b,c) values (with 95% CI) for left ventricular (LV) ejection fraction (a), global longitudinal strain (b), mitral valve E/A ratio (c) and left atrial (LA) emptying fraction (d), among women classified as being at low risk for pre‐eclampsia (PE) (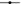), those at high risk for PE who did not develop hypertensive disorders of pregnancy (HDP) (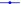) and those at high risk for PE who developed HDP (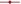), at 6–12 weeks, 6 months and 12 months postpartum. 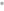
Statistically significant difference between groups.

After multivariable analysis, among high‐risk women, those with HDP demonstrated a persistently worse systolic profile throughout the first year postpartum, indicated by increased myocardial performance index and greater impairment in GLS, along with lower mitral annular plane systolic excursion, compared to those without HDP (Table S4). Additionally, the significant differences in LV‐EF observed at 6–12 weeks postpartum persisted for up to 6 months postpartum (Table S4).

### Diastolic function

At 6–12 weeks postpartum, compared with the low‐risk control group, the high‐risk‐with‐HDP group exhibited reduced diastolic function, indicated by impaired ventricular relaxation (a lower mitral valve (MV) E/A ratio), and worsened atrial function (decreased left atrial expansion index (LA‐EI) and LA emptying fraction, along with a higher minimum left atrial volume index (LA‐VImin)) (Figure 3c,d, Table 2). Similarly, the high‐risk‐with‐HDP group exhibited reduced diastolic function compared with the high‐risk‐without‐HDP group, as shown by lower MV lateral e′ velocity and LA‐EI (Table 2).

At 6 and 12 months postpartum, the high‐risk‐with‐HDP group continued to exhibit reduced diastolic function, as indicated by a reduced LA emptying fraction and LA‐EI, compared with the high‐risk‐without‐HDP group (Table 2). Multivariable analysis among high‐risk women revealed that the diastolic impairments observed at 6–12 weeks persisted at 12 months postpartum in women who developed HDP (Table S4). Advancing age was also associated with reduced diastolic function, as indicated by lower MV‐E/A ratio, MV lateral annular e′ velocity and higher maximum left atrial volume (LA‐VImax) as well as LA‐VImin (*P* < 0.05, data not shown). Moreover, increased weight was associated with reduced diastolic function, as evidenced by lower MV e′ septal annular velocity and higher values of E/e′ (*P* < 0.05, data not shown). Anthropometric and cardiovascular profiles of participants after excluding cases with new‐onset chronic hypertension and/or a previous history of HDP are presented in Appendix S3 and supported by data in Tables S5–S7.

### Temporal changes in maternal anthropometric and cardiovascular parameters from 6–12 weeks to 12 months postpartum

The high‐risk‐without‐HDP group demonstrated a significant decreasing trend in waist circumference and waist‐to‐hip ratio from 6–12 weeks to 12 months postpartum (*P* < 0.001), whereas the high‐risk‐with‐HDP group exhibited no significant improvement in these parameters between postpartum visits (Table S3). Similar findings were observed after excluding cases with new‐onset chronic hypertension and/or a previous history of HDP (Table S5). Additionally, from 6 to 12 weeks postpartum, the high‐risk‐without‐HDP group exhibited a downward trend in weight until 12 months postpartum (*P* = 0.03) and reduced weight gain from the first trimester to 12 months postpartum (*P* = 0.025), while the high‐risk‐with‐HDP group showed no significant change in these parameters during the same period (Table S3).

No significant changes in postpartum hemodynamics from 6–12 weeks to 12 months postpartum were observed in either of the two high‐risk groups (Tables S3 and S6). Regarding temporal changes in cardiac geometry, the high‐risk‐without‐HDP group exhibited an improvement in LV‐MI from 6–12 weeks to 6 months postpartum (*P* < 0.001), while this improvement was delayed until 12 months postpartum in those who developed HDP (*P* = 0.029; Table S4). Similar patterns were observed after excluding cases with new‐onset chronic hypertension (Table S6).

Regarding temporal changes in systolic function, the pattern differed between the two high‐risk groups: a significant improvement in LV‐EF and GLS was seen from 6 to 12 weeks to 6 months postpartum and this persisted at 12 months postpartum in the group without HDP, while this improvement was delayed and only became apparent at 12 months postpartum in those with HDP (Table S4). Similar findings were observed after excluding cases with new‐onset chronic hypertension and/or a previous history of HDP (Table S7).

Regarding diastolic functional parameters, from 6–12 weeks to 12 months postpartum, the high‐risk‐ without‐HDP group showed a significant improvement in key diastolic parameters (MV‐E/A ratio, LA‐VImin and E/e′ ratio). Moreover, this group exhibited a significant improvement in LA‐EF, as well as LA‐EI from 6–12 weeks to 6 months postpartum, whereas these improvements in the high‐risk‐with‐HDP group only became apparent at 12 months postpartum (Table S4). These findings were consistent after excluding cases with new‐onset chronic hypertension and/or a previous history of HDP (Table S7).

### Postpartum cardiovascular profile stratified by subtype of HDP

Compared with high‐risk women who did not develop HDP, those who developed gestational hypertension or late‐onset PE exhibited consistently higher MAP across all three postpartum visits (Figure S1a, Table S8). In addition, distinct patterns of the cardiovascular profile were observed among the HDP subtypes. The late‐onset PE group demonstrated reduced systolic function, as evidenced by greater impairment of GLS at both 6–12 weeks and 6 months postpartum compared with the high‐risk‐without‐HDP group. The gestational hypertension group showed evidence of structural changes, with significantly higher LV‐MI at both 6 and 12 months postpartum (Figure S1b, Table S8) and worse GLS at 12 months postpartum, compared with the high‐risk‐without‐HDP group.

Regarding temporal changes in the cardiovascular profile among different subtypes of HDP, no significant cardiovascular changes from 6–12 weeks to 12 months postpartum were observed, except that early‐onset PE and late‐onset PE groups exhibited improvement in systolic function (GLS for clinical visits comparison: *P* = 0.017 and *P* = 0.02, respectively) by 12 months postpartum, while the gestational‐hypertension group did not show this improvement (Table S8).

## DISCUSSION

### Principal findings

There are two main findings of this study. Firstly, from 6–12 weeks to 12 months postpartum, women at high risk of PE who did not develop HDP showed a downward trend in waist circumference, waist‐to‐hip ratio and weight, with reduced weight gain from the first trimester to 12 months postpartum. In contrast, those who developed HDP exhibited a static profile in these anthropometric measures over the same period. Secondly, regarding the cardiac recovery timeline in the postpartum period, the high‐risk‐without‐HDP group demonstrated earlier improvement in heart structure and function starting from 6–12 weeks, whereas these temporal cardiac improvements in the high‐risk‐with‐HDP group were often delayed and only become apparent at 12 months postpartum, a finding that persisted even after excluding cases with new‐onset chronic hypertension and/or a previous history of HDP.

### Results in the context of what is known

Women with a history of PE or eclampsia tend to exhibit a worse metabolic profile later in life compared with those with previous normotensive pregnancies^38^. This study revealed that women at high risk for PE who developed HDP showed no or little improvement in anthropometric measurements during the first year postpartum, compared with a more favorable anthropometric trajectory in women who did not develop HDP.

The persistent elevation in SVR, increased LV‐MI and impaired systolic and diastolic function observed in the high‐risk‐with‐HDP group over the postpartum period closely align with the high‐resistance, low‐output hemodynamics seen in cases with early‐onset PE. These findings are often present before pregnancy in women predisposed to HDP, reflecting underlying endothelial dysfunction and altered ventricular–arterial coupling^15^, and can persist 12–18 months postpartum, contributing to recurrent PE and long‐term CVD^16^. The findings of persistently high SVR and elevated LV‐MI in the high‐risk‐with‐HDP group were associated with endothelial dysfunction, impaired venous compliance and maladaptive arterial remodeling in the postpartum period. This is supported by the recommendations of the International Working Group on Maternal Hemodynamics, which emphasize that venous hemodynamics, capacitance and volume homeostasis are central determinants of cardiovascular adaptation and play a critical role in the pathophysiology of HDP^18^. Impairments in venous return and preload reserve, which are key features of the high‐resistance phenotype, may therefore contribute to the delayed normalization of MAP, GLS and diastolic indices observed in this study.

### Strengths and limitations

The main strength of this study lies in its prospective design, allowing tracking of longitudinal postnatal cardiovascular trajectories in high‐risk women at 6–12 weeks, 6 months and 12 months postpartum, in accordance with the AHA guidelines^18^, ^19^, and its examination of the relationship between maternal risk factors and cardiovascular function. We employed comprehensive and advanced techniques such as speckle‐tracking analysis to assess cardiac function.

There are, however, some limitations to this study. In the group of high‐risk women with HDP, only those with a diagnosis of PE or gestational hypertension were included in this study. The inclusion of women with chronic hypertension may have led to an overestimation of cardiovascular impairment. Of note, differences in the prevalence of chronic hypertension between different study populations may contribute to the variation in reported anthropometric and cardiovascular findings across studies. The limited number of participants using antihypertensive medication postpartum (one case at 6–12 weeks and one case at all three clinical visits) was not sufficient to yield adequate statistical power to be accounted for in the analysis. However, the underlying effect of medication is acknowledged as a limitation. In this study, the low‐risk group was assessed only at 6–12 weeks postpartum and therefore cannot serve as a longitudinal comparator for physiological postpartum recovery. Another limitation of the study is the lack of data on prepregnancy waist‐to‐hip ratio and prepregnancy hemodynamics, precluding an investigation of changes from the prepregnancy period to the postpartum period. We acknowledge that attrition (~25%) may introduce bias despite similar baseline characteristics between included and excluded participants, and that the relatively small HDP subgroup may have limited the stability of the fully adjusted models; multiple comparisons were addressed using the Holm–Bonferroni correction, with interpretation focusing primarily on cardiovascular parameters (e.g. MAP, SVR, LV‐MI and GLS).

### Clinical and research implications

This study highlights the suboptimal postpartum cardiometabolic trajectory beyond 6–12 weeks in women at high risk of PE, particularly those who develop HDP. Based on persistently suboptimal anthropometric and cardiovascular trajectories (waist circumference, waist‐to‐hip ratio, MAP, SVR, LV‐MI and GLS), we propose a structured postpartum follow‐up protocol, with baseline assessment between 6 and 12 weeks, reassessment at 6 months and evaluation of the recovery trajectory and long‐term cardiovascular risk stratification at 12 months. Given that LV dimensions and LV‐MI are typically smaller in the Asian population compared with the White and Afro‐Caribbean populations^39^, ^40^, the standard clinical cut‐off for cardiac hypertrophy (e.g. ≥ 95 g/m^2^) may not be directly applicable in Asian individuals. Referral to cardiology may be advisable for women who experience HDP and exhibit a persistently adverse cardiovascular profile in the postpartum period, characterized by a waist‐to‐hip ratio ≥ 0.85, MAP ≥ 90 mmHg and persistent increased LV‐MI (≥ 78 g/m^2^) and/or a reduced MV‐E/A ratio^39^, ^41^, ^42^. Further studies on the association between these parameters, using these thresholds, and key clinical outcomes, such as future CVD, are warranted. Additionally, most observed echocardiographic differences remained below established clinical thresholds, suggesting the presence of subclinical physiological alterations whose implications on long‐term cardiovascular outcomes remain to be determined. The proposed follow‐up pathway could be integrated into the existing postpartum care framework by synchronizing with routine newborn vaccination visits. Widespread implementation will be necessary to overcome potential barriers, such as disparities in access to echocardiography, resource allocation for extended follow‐up and challenges in improving patient engagement and retention in cardiovascular risk monitoring. Addressing these challenges would enable early identification of women at the highest risk for future CVD, ultimately facilitating timely interventions and enhancing long‐term maternal health outcomes.

### Conclusions

Women at high risk for PE, particularly those who develop HDP, exhibit distinctly suboptimal anthropometric and cardiovascular trajectories during the first year postpartum. The observed association between maternal risk factors (e.g. increased weight, advanced age, prior HDP) and an unfavorable cardiovascular profile underscores the need to extend anthropometric and cardiovascular screening and implement personalized interventions beyond the standard 6–12‐week postpartum visit in women who develop HDP. However, the feasibility and practicality of postpartum cardiovascular surveillance are limited by disparities in access to echocardiography, resource allocation for prolonged follow‐up and challenges in improving patient engagement in postpartum care.

## Supporting information


**Appendix S1** Protocols for echocardiographic measurements, including cardiac hemodynamics, geometry and function.
**Appendix S2** Outline of measurement protocol to obtain key echocardiographic parameters.
**Appendix S3** Anthropometric and cardiovascular profile of participants after excluding cases with newly‐onset chronic hypertension and/or previous history of hypertensive disorders of pregnancy (HDP).
**Table S1** Maternal characteristics of study population according to included and excluded cases.
**Table S2** Effect size for comparison of cardiovascular parameters among low‐risk, high‐risk‐without‐hypertensive‐disorders‐of‐pregnancy (HDP) and high‐risk‐with‐HDP groups at 6–12 weeks postpartum.
**Table S3** Estimated marginal means and effect size of anthropometric and hemodynamic parameters between women at high risk of pre‐eclampsia with and those without hypertensive disorders of pregnancy (HDP) at each clinical visit.
**Table S4** Estimated marginal means and effect size of cardiovascular parameters between women at high risk of pre‐eclampsia with and those without hypertensive disorders of pregnancy (HDP) at each clinical visit.
**Table S5** Sensitivity analysis of estimated marginal means and effect size of anthropometric parameters between women at high risk of pre‐eclampsia with and those without hypertensive disorders of pregnancy (HDP) at each clinical visit, stratified by exclusion criteria.
**Table S6** Sensitivity analysis of estimated marginal means and effect size of cardiac hemodynamics and geometry between women at high risk of pre‐eclampsia with and those without hypertensive disorders of pregnancy (HDP) at each clinical visit, stratified by exclusion criteria.
**Table S7** Sensitivity analysis of estimated marginal means and effect size of cardiac systolic and diastolic function between women at high risk of pre‐eclampsia with and those without hypertensive disorders of pregnancy (HDP) at each clinical visit, stratified by exclusion criteria.
**Table S8** Estimated marginal means of cardiovascular profile for women with different types of hypertensive disorder of pregnancy.
**Figure S1** Trajectory of mean arterial pressure (a) and mean left ventricular mass index (b) with 95% CI among four outcome groups in the postpartum (PN) period, including high risk without hypertensive disorders of pregnancy (HDP), gestational hypertension (GH), late‐onset pre‐eclampsia (PE) and early‐onset PE.

## Data Availability

The data that support the findings of this study are available from the corresponding author upon reasonable request.
