# Supplementary material for: Postpartum anthropometric and cardiovascular profile following hypertensive disorders of pregnancy: 12‐month follow‐up
Source: Ultrasound Obstet Gynecol. 2026 Sep 28;68(4):527–39. doi: 10.1002/uog.70313 (PMC13641731; doi:10.1002/uog.70313)
Supplement: Supplementary file 1 [file UOG-68-527-s001.docx]

**SUPPORTING INFORMATION**

**Appendix S1: Protocols for echocardiographic measurements, including cardiac hemodynamics, geometry and function**

Hemodynamic parameters, including stroke volume (SV), cardiac output (CO), and systemic vascular resistance (SVR), were assessed as previously reported^1^. LV mass and relative wall thickness (RWT) were obtained using M-mode in the parasternal long axis view. LV mass (LVM), and RWT were evaluated following an international guideline ^2^. LVM was indexed to body surface area to calculate the LVM index (LVMI). All the measurements were acquired during the diastolic period. LV volume, including LV end-diastolic volume (LVEDV) and LV end-systolic volume (LVESV), were measured using a Modified Simpson method, and LV ejection fraction (LVEF) was calculated. Global myocardial contractility was assessed by calculating the myocardial performance index (MPI). It was recorded as the average of the tissue Doppler-MPI measured at the septal and lateral sides of the mitral annulus. In the four-chamber view, the mitral annular plane systolic excursion (MAPSE) was obtained from M-mode recording. Parameters of diastolic function consist of mitral valve (MV) E velocity, A velocities and their ratio (E/A), tissue Doppler e’ velocities of the septal and lateral mitral annulus, the ratio of MV E velocity and mean e’ velocity (E/e’ ratio) and left atrial (LA) volume. Maximal left atrial volume (LAVmax) was measured using the Biplane modified Simpson method in the apical four-and two-chamber views at the end-systolic frame just before MV opening. Similarly, minimal left atrial volume (LAVmin) was measured at the end-diastolic frame just before MV closure. LAVmax and LAVmin were indexed to body surface area for LAVImax and LAVImin. LA emptying fraction (LAEF) and LA expansion index (LAEI) were calculated as previously reported^3^. Speckle-tracking analysis was performed in the four-chamber, two-chamber, and three-chamber views to calculate LV global longitudinal strain (GLS) ^2^.

**Appendix S2 Outline of measurement protocol to obtain key echocardiographic parameters.**

1. Left ventricular mass index

LV mass and relative wall thickness (RWT) were obtained using M-mode in the parasternal long axis view. LV mass (LVM), and RWT were evaluated following an international guideline ^1^. LVM was indexed to body surface area to calculate the LVM index (LVMI).

2. Myocardial performance index

Myocardial performance index (MPI) is recorded as the average of the tissue Doppler-MPI measured at the septal and lateral sides of the mitral annulus.

To measure the MPI by tissue Doppler imaging, time intervals are measured from the septal mitral annulus.

Isovolumic contraction time (ICT) is measured from the end of a′-wave (atrial-contraction wave) to the onset of s-wave (myocardial systolic wave);

The ejection time (ET) is measured from the onset to the end of s-wave (ET).

Isovolumic relaxation time (IRT) is measured between the end of the s-wave and the onset of the e′-wave (early-diastolic wave). The MPI is calculated as (ICT+IRT)/ET. An average MPI is calculated between MPI obtained at lateral and septal sides of mitral annulus.

3. Mitral annular plane systolic excursion

To measure the mitral annular plane systolic excursion (MAPSE), an M-mode cursor is placed at the septal and lateral portion of the mitral valve annulus in the four-chamber view. This will generate a wave form reflecting valvular motion during a cardiac cycle. The systolic excursion is quantified by measuring the highest (end-diastole) and lowest (end-systolic) point of the waveform.

4. Global longitudinal strain

Speckle-tracking analysis is performed in the four-chamber, two-chamber, and three-chamber views to calculate LV global longitudinal strain (GLS) ^1^.

1.	Lang RM, Badano LP, Mor-Avi V, Afilalo J, Armstrong A, Ernande L, et al. Recommendations for cardiac chamber quantification by echocardiography in adults: An update from the american society of echocardiography and the european association of cardiovascular imaging. *Eur Heart J Cardiovasc Imaging*. 2015;16:233-270

**Appendix S3 Anthropometric and cardiovascular profile of participants after excluding cases with newly-onset chronic hypertension and/or previous history of HDP**

After excluding cases with newly-onset chronic hypertension and/or a previous history of HDP, the high risk with HDP group showed a less favorable anthropometric and cardiovascular profile compared with the high risk without HDP group. More precisely, compared to high risk women without HDP, those high risk women who developed HDP demonstrated: (1) a significantly higher waist circumference and waist-to-hip ratio at both 6 and 12 months postpartum (Table S5); (2) persistently higher MAP and LVMI across the three postpartum visits (Table S6); (3) reduced systolic function, as shown by lower MAPSE and GLS at 6-12 weeks and at 6 months postpartum (Table S4); and (4) reduced diastolic function, as evidenced by lower MV E/A ratio, LAEF and LAEI at 12 months postpartum (Table S7).

**Table S1 Maternal characteristics of study population according to included and excluded cases.**

| Variables | Included cases (N=201) | Excluded cases (N=66) | P |
| --- | --- | --- | --- |
| Height (m) | 1.59 ±0.05 | 1.59 ± 0.05 | 0.413 |
| Parity | 1.00 (1.00, 1.00) | 1.00 (1.00, 2.00) | 0.314 |
| Family history of CVD (%) | 94 (46.77) | 25 (37.88) | 0.264 |
| Previous history of HDP (%) | 16 (7.96) | 5 (7.58) | 1.000 |
| Diabetes mellitus (%) | 2 (1.00) | 2 (3.03) | 0.550 |
| Risk ratio for first-trimester PE screening | 52.00 (28.00, 83.00) | 49.50 (24.00, 74.00) | 0.167 |
| Gestational diabetes mellitus (%) | 25 (12.44) | 12 (18.18) | 0.334 |
| Gestational age at delivery (weeks) | 38.14 (37.43, 39.29) | 38.29 (37.46, 39.25) | 0.509 |
| Fetal birthweight (grams) | 2885.00 (2595.00, 3178.75) | 3017.50 (2667.50, 3222.50) | 0.352 |
| Age at 6-12 weeks postpartum (years) | 35.59 ± 4.54 | 34.72 ± 4.46 | 0.179 |
| Weight at 6-12 weeks postpartum (kg) | 61.80 (56.10, 67.20) | 62.15 (54.10, 72.59) | 0.769 |
| BMI at 6-12 weeks postpartum (kg/ m^2^) | 24.57 (22.24, 26.50) | 24.88 (21.49, 28.60) | 0.752 |
| Waist circumference at 6-12 weeks postpartum (cm) | 86.92 ± 8.09 | 87.46 ±10.25 | 0.663 |
| Hip circumference at 6-12 weeks postpartum (cm) | 98.97 ± 7.31 | 99.49 ± 8.82 | 0.639 |
| Waist-to-hip ratio at 6-12 weeks postpartum | 0.88 ± 0.05 | 0.88± 0.05 | 0.947 |
| Mean arterial pressure at 6-12 weeks postpartum (mmHg) | 84.58 (79.33, 92.50) | 85.83 (79.81, 98.54) | 0.302 |

BMI: body mass index; CVD: cardiovascular disease; HDP: hypertensive disorders of pregnancy;

**Table S2 Effect size for comparison of cardiovascular parameters among low-risk, high risk without hypertensive disorder of pregnancy (HDP) and high risk with HDP groups at 6-12 weeks postpartum**

| **Variable** | **HR-non-HDP vs low-risk** | | **HR-HDP vs low-risk** | | **HR-HDP vs HR-non-HDP** | |
| --- | --- | --- | --- | --- | --- | --- |
|  | **Cohen's d (95% CI)** | **P value** | **Cohen's d (95% CI)** | **P value** | **Cohen's d (95% CI)** | **P value** |
| **Anthropometrics** |  |  |  |  |  |  |
| Weight (kg) | -0.19 (-0.59, 0.20) | 0.343 | 0.11 (-0.37, 0.58) | 0.655 | 0.30 (-0.05, 0.65) | 0.123 |
| Weight gain from first-trimester to postpartum (kg) | -0.18 (-0.40, 0.07) | 0.124 | -0.35 (-0.63, -0.10) | 0.012 | -0.19 (-0.39, 0.01) | 0.065 |
| Body mass index (kg/m^2^) | -0.11 (-0.51, 0.28) | 0.543 | 0.21 (-0.27, 0.68) | 0.367 | 0.32 (-0.03, 0.68) | 0.127 |
| Waist circumference (cm) | 0.35 (-0.05, 0.75) | 0.086 | 0.65 (0.16, 1.13) | 0.008 | 0.34 (-0.01, 0.70) | 0.086 |
| Hip circumference (cm) | -0.09 (-0.49, 0.30) | 0.648 | 0.12 (-0.36, 0.59) | 0.627 | 0.21 (-0.14, 0.57) | 0.264 |
| Waist-to-hip | 0.61 (0.21, 1.01) | 0.003 | 0.94 (0.44, 1.44) | <0.001 | 0.25 (-0.10, 0.61) | 0.142 |
| **Hemodynamics** |  |  |  |  |  |  |
| MAP (mmHg) | 0.31 (-0.08, 0.71) | 0.123 | 1.20 (0.69, 1.71) | <0.001 | 1.02 (0.65, 1.39) | <0.001 |
| SV (mL) | -0.95 (-1.36, -0.54) | <0.001 | -1.40 (-1.93, -0.87) | <0.001 | -0.25 (-0.61, 0.10) | 0.122 |
| CO (L/min) | -0.48 (-0.88, -0.08) | 0.006 | -0.46 (-0.94, 0.02) | 0.050 | 0.04 (-0.31, 0.40) | 0.807 |
| Heart rate (beats per min) | 0.43 (0.03, 0.83) | 0.026 | 0.73 (0.24, 1.22) | 0.003 | 0.27 (-0.09, 0.63) | 0.140 |
| SVR (dynes⋅sec/cm⁵) | 0.71 (0.30, 1.11) | <0.001 | 1.23 (0.71, 1.75) | <0.001 | 0.49 (0.13, 0.85) | 0.015 |
| **Geometry** |  |  |  |  |  |  |
| LVMI (g/m^2^) | 0.57 (0.17, 0.98) | 0.004 | 1.20 (0.69, 1.71) | <0.001 | 0.57 (0.21, 0.92) | 0.002 |
| RWT | 0.63 (0.23, 1.03) | <0.001 | 1.11 (0.61, 1.62) | <0.001 | 0.53 (0.18, 0.89) | 0.009 |
| LVEDV (mL) | 0.11 (-0.29, 0.52) | 0.552 | 0.52 (0.03, 1.01) | 0.037 | 0.36 (-0.01, 0.72) | 0.046 |
| LVESV (mL) | 0.55 (0.14, 0.96) | 0.002 | 1.20 (0.67, 1.72) | <0.001 | 0.54 (0.17, 0.90) | 0.005 |
| **Systolic function** |  |  |  |  |  |  |
| LV EF (%) | -1.04 (-1.46, -0.61) | <0.001 | -1.70 (-2.26, -1.14) | <0.001 | -0.55 (-0.91, -0.18) | 0.003 |
| MPI | 0.58 (0.15, 1.01) | 0.003 | 1.16 (0.63, 1.70) | <0.001 | 0.57 (0.21, 0.93) | 0.004 |
| MAPSE (cm) | 0.23 (-0.18, 0.64) | 0.221 | -0.59 (-1.09, -0.10) | 0.014 | -0.79 (-1.16, -0.43) | <0.001 |
| GLS (%) | 0.67 (0.25, 1.08) | <0.001 | 1.63 (1.07, 2.19) | <0.001 | 0.64 (0.27, 1.01) | <0.001 |
| **Diastolic function** |  |  |  |  |  |  |
| MV EA | -0.55 (-0.95, -0.15) | 0.044 | -0.82 (-1.31, -0.32) | 0.003 | -0.41 (-0.78, -0.05) | 0.029 |
| MV septal e' velocity | -0.05 (-0.47, 0.37) | 0.813 | -0.41 (-0.91, 0.09) | 0.096 | -0.36 (-0.72, -0.00) | 0.061 |
| MV lateral e' velocity | -0.03 (-0.45, 0.39) | 0.890 | -0.47 (-0.97, 0.03) | 0.057 | -0.45 (-0.81, -0.08) | 0.026 |
| E/e' | 0.07 (-0.36, 0.49) | 0.746 | 0.26 (-0.25, 0.76) | 0.321 | 0.18 (-0.20, 0.55) | 0.337 |
| LA VmaxI (mL/m^2^) | 0.13 (-0.29, 0.56) | 0.468 | 0.34 (-0.16, 0.84) | 0.148 | 0.20 (-0.15, 0.56) | 0.292 |
| LA VminI (mL/m^2^) | 1.20 (0.75, 1.64) | <0.001 | 1.53 (0.97, 2.10) | <0.001 | 0.46 (0.10, 0.82) | 0.029 |
| LA EF | -2.35 (-2.85, -1.85) | <0.001 | -2.44 (-3.09, -1.79) | <0.001 | -0.54 (-0.90, -0.18) | 0.013 |
| LA EI | -2.69 (-3.21, -2.17) | <0.001 | -2.75 (-3.44, -2.07) | <0.001 | -0.48 (-0.84, -0.12) | 0.017 |

CO: Cardiac output; LV EF: Left ventricular ejection fraction; GLS: Global longitudinal strain; LAEI: Left atrial expansion Index; LAEF: Left atrial emptying fraction; LAVImax: Maximum left atrial volume index; LAVImin: Minimum left atrial volume index; LVMI: Left ventricular mass index; LVEDV: Left ventricular end-diastolic volume; LVESV: Left ventricular end-systolic volume; MAPSE: Mitral annular plane systolic excursion; MAP: Mean arterial pressure; MPI: Myocardial performance index; RWT: Relative wall thickness; SV: Stroke volume; SVR: Systemic vascular resistance

**Table S3 Estimated marginal means and effect size of anthropometric and hemodynamic parameters between women at high risk of pre-eclampsia with and without hypertensive disorder of pregnancy (HDP) at each study visit.**

| **Visit** | **Non-HDP** | **HDP** | **Cohen's d**  **(95% CI)** | **P (between groups)** | **Visit** | **Non-HDP** | **HDP** | **Cohen's d (95% CI)** | **P (between groups)** |
| --- | --- | --- | --- | --- | --- | --- | --- | --- | --- |
| **Anthropometrics** | | | | | | | | | |
| **Weight (kg)** |  |  |  |  | **BMI (kg/m^2^)** |  |  |  |  |
| 6-12 weeks | 57.0 (48.9 to 65.1) | 59.6 (51.4 to 67.9) | 1.76 (-0.48 to 4.01) | 0.126 | 6-12 weeks | 22.43 (19.13 to 25.74) | 23.55 (20.20 to 26.91) | 1.25 (-0.25 to 2.75) | 0.104 |
| 6 months | 56.6 (48.4 to 64.7) | 60.2 (51.9 to 68.5) | 2.50 (0.11 to 4.88) | 0.042 | 6 months | 22.25 (18.94 to 25.57) | 23.80 (20.42 to 27.18) | 1.70 (0.19 to 3.20) | 0.028 |
| 12 months | 56.1 (47.9 to 64.3) | 60.5 (52.1 to 68.9) | 2.97 (0.40 to 5.55) | 0.025 | 12 months | 22.06 (18.73 to 25.39) | 23.91 (20.48 to 27.34) | 2.03 (0.52 to 3.54) | 0.009 |
| *P (between visits)* | 0.03 # | 0.308 |  |  | *P (between visits)* | 0.052 | 0.356 |  |  |
| **Weight gain from first-trimester to postpartum (kg)** |  |  |  |  | **Waist circumference (cm)** |  |  |  |  |
| 6-12 weeks | 2.7 (-0.8 to 6.2) | 1.5 (-2.1 to 5.1) | -0.83 (-1.87 to 0.21) | 0.119 | 6-12 weeks | 89.43 (85.93 to 92.94) | 89.93 (86.31 to 93.56) | 0.12 (-0.30 to 0.54) | 0.581 |
| 6 months | 2.2 (-1.3 to 5.8) | 2.1 (-1.5 to 5.7) | -0.10 (-1.11 to 0.91) | 0.846 | 6 months | 88.42 (84.91 to 91.92) | 90.34 (86.72 to 93.96) | 0.46 (0.03 to 0.88) | 0.036 |
| 12 months | 1.8 (-1.8 to 5.3) | 2.3 (-1.3 to 6.0) | 0.38 (-0.76 to 1.52) | 0.511 | 12 months | 86.62 (83.07 to 90.18) | 88.58 (84.81 to 92.35) | 0.47 (-0.04 to 0.97) | 0.073 |
| *P (between visits)* | 0.025 # | 0.324 |  |  | *P (between visits)* | <0.001 *#† | 0.157 |  |  |
| **HC (cm)** |  |  |  |  | **Wasit-to-hip ratio** |  |  |  |  |
| 6-12 weeks | 99.64 (97.01 to 102.26) | 98.97 (96.26 to 101.68) | -0.25 (-0.73 to 0.23) | 0.314 | 6-12 weeks | 0.90 (0.86 to 0.94) | 0.91 (0.87 to 0.95) | 0.25 (-0.22 to 0.72) | 0.294 |
| 6 months | 99.16 (96.53 to 101.79) | 98.58 (95.87 to 101.29) | -0.21 (-0.70 to 0.28) | 0.394 | 6 months | 0.89 (0.85 to 0.93) | 0.92 (0.88 to 0.96) | 0.58 (0.12 to 1.05) | 0.015 |
| 12 months | 98.72 (96.06 to 101.37) | 97.18 (94.38 to 99.97) | -0.57 (-1.13 to -0.01) | 0.047 | 12 months | 0.88 (0.84 to 0.91) | 0.91 (0.87 to 0.95) | 0.86 (0.38 to 1.34) | <0.001 |
| *P (between visits*) | 0.071 | 0.032 # |  |  | *P (between visits)* | <0.001 #† | 0.8 |  |  |
| **Hemodynamics** | | | | | | | | | |
| **MAP (mmHg)** |  |  |  |  | **SV (mL)** |  |  |  |  |
| 6-12 weeks | 85.2 (78.2 to 92.2) | 93.2 (86.1 to 100.3) | 1.72 (1.05 to 2.38) | <0.001 | 6-12 weeks | 63.22 (55.96 to 70.48) | 59.80 (52.30 to 67.31) | -0.42 (-0.89 to 0.04) | 0.074 |
| 6 months | 85.0 (78.0 to 92.0) | 92.9 (85.8 to 100.0) | 1.69 (1.02 to 2.36) | <0.001 | 6 months | 62.98 (55.72 to 70.24) | 60.98 (53.49 to 68.48) | -0.25 (-0.71 to 0.21) | 0.294 |
| 12 months | 85.6 (78.6 to 92.7) | 93.2 (86.0 to 100.3) | 1.60 (0.92 to 2.29) | <0.001 | 12 months | 62.89 (55.60 to 70.17) | 60.17 (52.60 to 67.73) | -0.34 (-0.82 to 0.14) | 0.171 |
| *P (between visits)* | 0.523 | 0.959 |  |  | *P (between visits)* | 0.946 | 0.796 |  |  |
| **CO (L/min)** |  |  |  |  | **Heart rate** |  |  |  |  |
| 6-12 weeks | 3.96 (3.43 to 4.48) | 3.94 (3.39 to 4.49) | -0.02 (-0.44 to 0.39) | 0.914 | 6-12 weeks | 63.06 (56.36 to 69.77) | 66.16 (59.22 to 73.11) | 0.40 (-0.05 to 0.86) | 0.083 |
| 6 months | 4.09 (3.56 to 4.61) | 4.04 (3.49 to 4.58) | -0.07 (-0.48 to 0.35) | 0.75 | 6 months | 64.91 (58.20 to 71.61) | 66.79 (59.86 to 73.73) | 0.25 (-0.21 to 0.70) | 0.289 |
| 12 months | 4.06 (3.54 to 4.59) | 4.04 (3.49 to 4.59) | -0.03 (-0.47 to 0.40) | 0.875 | 12 months | 64.33 (57.60 to 71.06) | 67.16 (60.15 to 74.16) | 0.37 (-0.10 to 0.84) | 0.128 |
| *P (between visits)* | 0.308 | 0.774 |  |  | *P (between visits*) | 0.154 | 0.849 |  |  |
| **SVR** |  |  |  |  | **SVRI** |  |  |  |  |
| 6-12 weeks | 1797.69 (1565.73 to 2029.65) | 1955.05 (1714.05 to 2196.04) | 0.56 (0.12 to 1.00) | 0.014 | 6-12 weeks | 1147.97 (967.40 to 1328.55) | 1209.00 (1022.53 to 1395.48) | 61.03 (-27.68 to 149.74) | 0.179 |
| 6 months | 1760.73 (1528.76 to 1992.71) | 1927.77 (1687.23 to 2168.30) | 0.60 (0.16 to 1.03) | 0.008 | 6 months | 1129.91 (949.41 to 1310.42) | 1188.56 (1002.23 to 1374.90) | 58.65 (-29.31 to 146.60) | 0.193 |
| 12 months | 1784.66 (1551.88 to 2017.43) | 1946.24 (1702.99 to 2189.49) | 0.58 (0.12 to 1.04) | 0.015 | 12 months | 1155.30 (974.54 to 1336.06) | 1200.11(1012.29 to 1387.94) | 44.81 (-46.28 to 135.90) | 0.336 |
| *P (between visits)* | 0.577 | 0.905 |  |  | *P (between visits*) | 0.52 | 0.872 |  |  |

Weight was adjusted by clinical visits (study timepoints), study group, age, height, smoking, family history of CVD, parity, history of previous HDP, aspirin prophylaxis during pregnancy, gestational diabetes mellitus, any breastfeeding and the interaction between clinical visits and study group; BMI was adjusted by clinical visits (study timepoints), study group, age, smoking, family history of CVD, parity, history of previous HDP, aspirin prophylaxis during pregnancy, gestational diabetes mellitus, any breastfeeding and the interaction between clinical visits and study group; others variables were adjusted by clinical visits (study timepoints), study group, age, height, weight, smoking, family history of CVD, parity, history of previous HDP, aspirin prophylaxis during pregnancy, gestational diabetes mellitus, any breastfeeding and the interaction between clinical visits and study group. BMI: Body mass index; CO: Cardiac output; HC: Hip circumference; GLS: Global longitudinal strain; MAP: Mean arterial pressure; SV: Stroke volume; SVR: Systemic vascular resistance; SVRI: Systemic vascular resistance index;

*: significant difference between 6-12 weeks and 6 months;

#: significant difference between 6-12 weeks and 12 months

†: significant difference between 6 months and 12 months

**Table S4 Estimated marginal means and effect size of cardiovascular parameters between women at high risk of pre-eclampsia with and without hypertensive disorder of pregnancy (HDP) at each study visit.**

| **Visit** | **Non-HDP** | **HDP** | **Cohen's d**  **(95% CI)** | **P (between groups)** | **Visit** | **Non-HDP** | **HDP** | **Cohen's d (95% CI)** | **P (between groups)** |
| --- | --- | --- | --- | --- | --- | --- | --- | --- | --- |
| **Geometry** | | | | | | | | | |
| **LVMI (g/m^2^)** |  |  |  |  | **RWT (cm)** |  |  |  |  |
| 6-12 weeks | 66.51 (58.67 to 74.35) | 72.47 (64.36 to 80.58) | 0.75 (0.26 to 1.24) | 0.003 | 6-12 weeks | 0.29 (0.25 to 0.33) | 0.33 (0.28 to 0.37) | 0.63 (0.22 to 1.04) | 0.003 |
| 6 months | 63.11 (55.27 to 70.95) | 68.71 (60.61 to 76.82) | 0.71 (0.22 to 1.20) | 0.005 | 6 months | 0.29 (0.25 to 0.33) | 0.31 (0.27 to 0.35) | 0.36 (-0.06 to 0.77) | 0.093 |
| 12 months | 62.26 (54.42 to 70.10) | 68.18 (60.07 to 76.28) | 0.75 (0.26 to 1.24) | 0.003 | 12 months | 0.28 (0.24 to 0.32) | 0.32 (0.28 to 0.36) | 0.70 (0.27 to 1.13) | 0.002 |
| *P (between visits)* | <0.001 *# | 0.029 # |  |  | *P (between visits)* | 0.217 | 0.299 |  |  |
| **LVEDV (mL)** |  |  |  |  | **LVESV (mL)** |  |  |  |  |
| 6-12 weeks | 78.62 (69.59 to 87.64) | 81.70 (72.44 to 90.96) | 0.39 (-0.15 to 0.94) | 0.162 | 6-12 weeks | 32.35 (27.81 to 36.90) | 35.12 (30.46 to 39.79) | 0.68 (0.14 to 1.21) | 0.014 |
| 6 months | 75.04 (66.00 to 84.08) | 79.54 (70.28 to 88.79) | 0.57 (0.02 to 1.12) | 0.043 | 6 months | 30.17 (25.62 to 34.72) | 33.13 (28.46 to 37.79) | 0.72 (0.18 to 1.26) | 0.009 |
| 12 months | 75.72 (66.65 to 84.78) | 76.30 (67.00 to 85.59) | 0.07 (-0.49 to 0.64) | 0.797 | 12 months | 29.66 (25.09 to 34.22) | 30.72 (26.03 to 35.40) | 0.26 (-0.29 to 0.81) | 0.358 |
| *P (between visits)* | <0.001 *# | 0.013 # |  |  | *P (between visits)* | <0.001 *# | 0.002 # |  |  |
| **Systolic function** | | | | | | | | | |
| **LVEF** |  |  |  |  | **MPI** |  |  |  |  |
| 6-12 weeks | 0.58 (0.56 to 0.61) | 0.56 (0.53 to 0.60) | -0.48 (-0.88 to -0.07) | 0.022 | 6-12 weeks | 0.37 (0.34 to 0.40) | 0.39 (0.36 to 0.43) | 0.53 (0.16 to 0.90) | 0.006 |
| 6 months | 0.60 (0.57 to 0.63) | 0.58 (0.55 to 0.61) | -0.41 (-0.81 to -0.01) | 0.048 | 6 months | 0.36 (0.33 to 0.39) | 0.38 (0.35 to 0.42) | 0.53 (0.16 to 0.90) | 0.005 |
| 12 months | 0.61 (0.58 to 0.64) | 0.59 (0.56 to 0.62) | -0.40 (-0.82 to 0.02) | 0.064 | 12 months | 0.34 (0.31 to 0.37) | 0.37 (0.33 to 0.40) | 0.45 (0.07 to 0.84) | 0.023 |
| *P (between visits)* | <0.001 *# | 0.0270 # |  |  | *P (between visits)* | 0.0017 # | 0.064 |  |  |
| **MAPSE (cm)** |  |  |  |  | **GLS** |  |  |  |  |
| 6-12 weeks | 1.54 (1.42 to 1.65) | 1.38 (1.26 to 1.51) | -1.00 (-1.42 to -0.57) | <0.001 | 6-12 weeks | -0.25 (-0.26 to -0.24) | -0.24 (-0.25 to -0.22) | 0.81 (0.37 to 1.26) | <0.001 |
| 6 months | 1.55 (1.43 to 1.67) | 1.42 (1.30 to 1.54) | -0.82 (-1.24 to -0.39) | <0.001 | 6 months | -0.26 (-0.27 to -0.24) | -0.24 (-0.26 to -0.23) | 0.97 (0.52 to 1.42) | <0.001 |
| 12 months | 1.53 (1.41 to 1.65) | 1.45 (1.32 to 1.58) | -0.50 (-0.95 to -0.05) | 0.033 | 12 months | -0.26 (-0.27 to -0.24) | -0.25 (-0.26 to -0.23) | 0.52 (0.06 to 0.99) | 0.03 |
| *P (between visits)* | 0.617 | 0.164 |  |  | *P (between visits)* | <0.001 *# | 0.005 # |  |  |
| **Diastolic function** | | | | | | | | | |
| **MV E/A** |  |  |  |  | **MV septal e'** |  |  |  |  |
| 6-12 weeks | 1.40 (1.14 to 1.67) | 1.25 (0.98 to 1.52) | -0.74 (-1.32 to -0.16) | 0.013 | 6-12 weeks | 11.04 (9.67 to 12.41) | 10.40 (8.98 to 11.82) | -0.43 (-0.91 to 0.04) | 0.074 |
| 6 months | 1.46 (1.19 to 1.72) | 1.35 (1.07 to 1.62) | -0.53 (-1.14 to 0.07) | 0.087 | 6 months | 11.33 (9.96 to 12.70) | 10.98 (9.57 to 12.40) | -0.24 (-0.71 to 0.23) | 0.324 |
| 12 months | 1.50 (1.23 to 1.77) | 1.34 (1.06 to 1.62) | -0.79 (-1.48 to -0.11) | 0.024 | 12 months | 11.46 (10.08 to 12.83) | 10.93 (9.50 to 12.35) | -0.36 (-0.85 to 0.13) | 0.15 |
| *P (between visits)* | 0.005 # | 0.112 |  |  | *P (between visits)* | 0.074 | 0.154 |  |  |
| **MV lateral e'** |  |  |  |  | **E/e’** |  |  |  |  |
| 6-12 weeks | 14.05 (12.26 to 15.84) | 12.98 (11.14 to 14.82) | -0.72 (-1.32 to -0.12) | 0.02 | 6-12 weeks | 5.93 (5.00 to 6.86) | 6.15 (5.20 to 7.11) | 0.25 (-0.28 to 0.77) | 0.359 |
| 6 months | 14.16 (12.37 to 15.96) | 13.53 (11.70 to 15.36) | -0.43 (-1.00 to 0.14) | 0.142 | 6 months | 6.19 (5.26 to 7.12) | 6.45 (5.49 to 7.41) | 0.29 (-0.22 to 0.80) | 0.262 |
| 12 months | 13.92 (12.12 to 15.71) | 13.56 (11.71 to 15.40) | -0.23 (-0.82 to 0.35) | 0.432 | 12 months | 6.25 (5.32 to 7.19) | 6.40 (5.44 to 7.37) | 0.17 (-0.35 to 0.70) | 0.525 |
| *P (between visits)* | 0.434 | 0.19 |  |  | *P (between visits)* | 0.015 # | 0.318 |  |  |
| **LAVImax (mL/m2)** |  |  |  |  | **LAVImin (mL/m^2^)** |  |  |  |  |
| 6-12 weeks | 28.77 (24.69 to 32.84) | 29.78 (25.56 to 34.00) | 0.22 (-0.24 to 0.67) | 0.349 | 6-12 weeks | 16.61 (14.03 to 19.19) | 18.08 (15.41 to 20.76) | 0.49 (0.04 to 0.95) | 0.034 |
| 6 months | 28.17 (24.09 to 32.24) | 28.81 (24.59 to 33.03) | 0.14 (-0.32 to 0.60) | 0.552 | 6 months | 15.88 (13.30 to 18.46) | 16.90 (14.23 to 19.57) | 0.34 (-0.11 to 0.80) | 0.139 |
| 12 months | 29.94 (25.86 to 34.02) | 30.70 (26.43 to 34.96) | 0.16 (-0.32 to 0.64) | 0.51 | 12 months | 15.48 (12.90 to 18.07) | 17.28 (14.57 to 19.98) | 0.60 (0.12 to 1.08) | 0.015 |
| *P (between visits)* | 0.015 † | 0.225 |  |  | *P (between visits)* | 0.015 # | 0.2 |  |  |
| **LAEF** |  |  |  |  | **LAEI** |  |  |  |  |
| 6-12 weeks | 0.42 (0.39 to 0.45) | 0.39 (0.36 to 0.43) | -0.47 (-0.83 to -0.10) | 0.014 | 6-12 weeks | 0.76 (0.64 to 0.87) | 0.68 (0.56 to 0.80) | -0.37 (-0.73 to -0.00) | 0.05 |
| 6 months | 0.43 (0.40 to 0.47) | 0.41 (0.38 to 0.45) | -0.37 (-0.74 to -0.00) | 0.051 | 6 months | 0.80 (0.69 to 0.92) | 0.76 (0.64 to 0.88) | -0.20 (-0.57 to 0.16) | 0.277 |
| 12 months | 0.48 (0.45 to 0.52) | 0.44 (0.40 to 0.48) | -0.77 (-1.16 to -0.38) | <0.001 | 12 months | 0.98 (0.87 to 1.10) | 0.83 (0.71 to 0.96) | -0.73 (-1.12 to -0.35) | <0.001 |
| *P (between visits)* | <0.001 *#† | 0.002 # |  |  | *P (between visits)* | <0.001 *#† | 0.006# |  |  |

Adjusted by clinical visits (study timepoints), study group, age, height, weight, smoking, family history of CVD, parity, history of previous HDP, aspirin prophylaxis during pregnancy, gestational diabetes mellitus, any breastfeeding and the interaction between clinical visits and study group.

BMI: Body mass index; CO: Cardiac output; HC: Hip circumference; LV EF: Left ventricular ejection fraction; GLS: Global longitudinal strain; LAEI: Left atrial expansion Index; LAEF: Left atrial emptying fraction; LAVImax: Maximum left atrial volume index; LAVImin: Minimum left atrial volume index; LVMI: Left ventricular mass index; LVEDV: Left ventricular end-diastolic volume; LVESV: Left ventricular end-systolic volume; MAPSE: Mitral annular plane systolic excursion; MAP: Mean arterial pressure; MPI: Myocardial performance index; MV: Mitral valve; RWT: Relative wall thickness; WC: Waist circumference; WHR: Waist-to-hip ratio; SV: Stroke volume; SVR: Systemic vascular resistance

*: significant difference between 6-12 weeks and 6 months;

#: significant difference between 6-12 weeks and 12 months

†: significant difference between 6 months and 12 months

**Table S5 Sensitivity analysis of estimated marginal means and effect size of anthropometric parameters between women at high risk of pre-eclampsia with and without hypertensive disorder of pregnancy (HDP) at each study visit, stratified by exclusion criteria.**

| **Visit** | **Exclusion of participants with newly-onset CHT** | | | | | **Exclusion of participants with previous history of HDP** | | | | | **Exclusion of participants with newly-onset CHT and previous history of HDP** | | | | |
| --- | --- | --- | --- | --- | --- | --- | --- | --- | --- | --- | --- | --- | --- | --- | --- |
|  | **Non-HDP** | **HDP** | **EMM (95%CI)** | **Cohen's d (95%)** | **P** | **Non-HDP** | **HDP** | **EMM (95%CI)** | **Cohen's d (95%)** | **P** | **Non-HDP** | **HDP** | **EMM (95%CI)** | **Cohen's d (95%)** | **P** |
| **Weight (kg)** | | | | | | | | | | | | | | | |
| **6-12 weeks** | 85.5 (79.0 to 92.0) | 93.4 (86.7 to 100.1) | 7.9 (4.8 to 10.9) | 1.69 (1.03 to 2.34) | **<0.001** | 59.0 (51.2 to 66.8) | 61.3 (53.1 to 69.5) | 2.3 (-1.3 to 5.9) | 1.51 (-0.87 to 3.89) | 0.214 | 59.6 (52.1 to 67.1) | 61.3 (53.5 to 69.2) | 1.7 (-1.9 to 5.3) | 1.13 (-1.23 to 3.48) | 0.35 |
| **6 months** | 85.4 (78.8 to 91.9) | 92.6 (85.9 to 99.3) | 7.2 (4.1 to 10.3) | 1.54 (0.89 to 2.20) | **<0.001** | 58.6 (50.7 to 66.5) | 62.2 (54.0 to 70.5) | 3.7 (-0.2 to 7.5) | 2.42 (-0.11 to 4.96) | 0.063 | 59.2 (51.7 to 66.7) | 62.3 (54.4 to 70.3) | 3.2 (-0.7 to 7.1) | 2.06 (-0.49 to 4.61) | 0.115 |
| **12 months** | 85.9 (79.4 to 92.4) | 92.6 (85.8 to 99.3) | 6.7 (3.5 to 9.8) | 1.43 (0.75 to 2.11) | **<0.001** | 58.1 (50.2 to 66.0) | 62.5 (54.1 to 70.9) | 4.4 (0.3 to 8.6) | 2.93 (0.18 to 5.68) | **0.039** | 58.7 (51.1 to 66.3) | 62.6 (54.5 to 70.8) | 3.9 (-0.4 to 8.2) | 2.57 (-0.23 to 5.37) | 0.075 |
| **P (between visits)** | 0.662 | 0.7 |  |  |  | 0.0618 | 0.11 |  |  |  | 0.056 | 0.129 |  |  |  |
| **Weight gain from first-trimester to postpartum (kg)** | | | | | | | | | | | | | | | |
| **6-12 weeks** | 3.0 (-0.6 to 6.6) | 1.7 (-2.0 to 5.4) | -1.3 (-2.9 to 0.4) | -0.84 (-1.94 to 0.25) | 0.133 | 1.8 (-1.7 to 5.3) | 0.3 (-3.3 to 3.9) | -1.5 (-3.2 to 0.2) | -0.99 (-2.10 to 0.13) | 0.086 | 1.9 (-1.6 to 5.4) | 0.4 (-3.3 to 4.1) | -1.5 (-3.3 to 0.3) | -0.97 (-2.13 to 0.19) | 0.102 |
| **6 months** | 2.5 (-1.1 to 6.1) | 2.4 (-1.3 to 6.1) | -0.1 (-1.7 to 1.5) | -0.06 (-1.11 to 0.99) | 0.917 | 1.4 (-2.1 to 4.9) | 1.3 (-2.3 to 4.9) | -0.1 (-1.7 to 1.5) | -0.08 (-1.16 to 1.00) | 0.888 | 1.5 (-2.0 to 5.0) | 1.4 (-2.3 to 5.1) | -0.1 (-1.8 to 1.7) | -0.04 (-1.17 to 1.08) | 0.94 |
| **12 months** | 2.0 (-1.6 to 5.6) | 2.5 (-1.2 to 6.3) | 0.5 (-1.3 to 2.3) | 0.35 (-0.84 to 1.54) | 0.567 | 0.9 (-2.6 to 4.4) | 1.5 (-2.2 to 5.2) | 0.6 (-1.2 to 2.5) | 0.42 (-0.80 to 1.64) | 0.503 | 1.0 (-2.5 to 4.5) | 1.7 (-2.1 to 5.5) | 0.7 (-1.3 to 2.6) | 0.45 (-0.83 to 1.72) | 0.493 |
| **P (between visits)** | 0.0254# | 0.324 |  |  |  | 0.057 | 0.113 |  |  |  | 0.044 # | 0.1334 |  |  |  |
| **Waist circumference (cm)** | | | | | | | | | | | | | | | |
| **6-12 weeks** | 89.20 (85.66 to 92.74) | 89.73 (86.03 to 93.44) | 0.53 (-1.35 to 2.42) | 0.13 (-0.33 to 0.58) | 0.579 | 88.16 (84.81 to 91.51) | 88.05 (84.45 to 91.65) | -0.11 (-2.02 to 1.80) | -0.03 (-0.49 to 0.43) | 0.912 | 88.07 (84.70 to 91.44) | 88.13 (84.47 to 91.79) | 0.06 (-1.95 to 2.07) | 0.01 (-0.48 to 0.50) | 0.955 |
| **6 months** | 88.12 (84.58 to 91.66) | 90.48 (86.78 to 94.17) | 2.36 (0.49 to 4.23) | 0.57 (0.12 to 1.02) | **0.015** | 87.06 (83.70 to 90.41) | 89.40 (85.80 to 93.01) | 2.35 (0.41 to 4.28) | 0.56 (0.10 to 1.03) | **0.019** | 86.89 (83.52 to 90.26) | 89.67 (86.01 to 93.33) | 2.78 (0.77 to 4.79) | 0.68 (0.19 to 1.17) | **0.007** |
| **12 months** | 86.16 (82.58 to 89.75) | 89.07 (85.21 to 92.93) | 2.91 (0.69 to 5.12) | 0.70 (0.17 to 1.24) | **0.011** | 85.18 (81.79 to 88.58) | 87.93 (84.19 to 91.67) | 2.75 (0.51 to 4.98) | 0.66 (0.12 to 1.20) | **0.017** | 84.84 (81.43 to 88.25) | 88.20 (84.42 to 91.98) | 3.36 (1.08 to 5.65) | 0.82 (0.26 to 1.38) | **0.005** |
| **P (between visits)** | <0.001 *#† | 0.342 |  |  |  | <0.001 *#† | 0.231 |  |  |  | <0.001 *#† | 0.214 |  |  |  |
| **Waist-to-hip ratio** | | | | | | | | | | | | | | | |
| **6-12 weeks** | 0.90 (0.86 to 0.94) | 0.91 (0.86 to 0.95) | 0.01 (-0.01 to 0.03) | 0.15 (-0.35 to 0.65) | 0.55 | 0.89 (0.86 to 0.93) | 0.90 (0.86 to 0.94) | 0.00 (-0.02 to 0.02) | 0.04 (-0.46 to 0.54) | 0.87 | 0.89 (0.86 to 0.93) | 0.89 (0.85 to 0.93) | -0.00 (-0.02 to 0.02) | -0.00 (-0.54 to 0.53) | 0.988 |
| **6 months** | 0.89 (0.85 to 0.93) | 0.92 (0.87 to 0.96) | 0.02 (0.00 to 0.04) | 0.56 (0.06 to 1.06) | **0.03** | 0.89 (0.85 to 0.92) | 0.91 (0.87 to 0.95) | 0.03 (0.01 to 0.05) | 0.63 (0.13 to 1.13) | **0.015** | 0.89 (0.85 to 0.92) | 0.91 (0.87 to 0.95) | 0.03 (0.00 to 0.05) | 0.60 (0.07 to 1.14) | **0.028** |
| **12 months** | 0.88 (0.84 to 0.92) | 0.92 (0.88 to 0.96) | 0.04 (0.02 to 0.07) | 1.05 (0.53 to 1.58) | **<0.001** | 0.87 (0.83 to 0.91) | 0.91 (0.87 to 0.95) | 0.04 (0.02 to 0.06) | 0.97 (0.46 to 1.49) | **<0.001** | 0.87 (0.83 to 0.91) | 0.91 (0.87 to 0.95) | 0.04 (0.02 to 0.07) | 1.06 (0.51 to 1.61) | **<0.001** |
| **P (between visits)** | <0.001 #† | 0.39 |  |  |  | <0.001 #† | 0.1866 |  |  |  | <0.001 #† | 0.161 |  |  |  |

Weight was adjusted by clinical visits (study timepoints), study group, age, height, smoking, family history of CVD, parity, history of previous HDP, aspirin prophylaxis during pregnancy, gestational diabetes mellitus, any breastfeeding and the interaction between clinical visits and study group; BMI was adjusted by clinical visits (study timepoints), study group, age, smoking, family history of CVD, parity, history of previous HDP, aspirin prophylaxis during pregnancy, gestational diabetes mellitus, any breastfeeding and the interaction between clinical visits and study group; others variables were adjusted by clinical visits (study timepoints), study group, age, height, weight, smoking, family history of CVD, parity, history of previous HDP, aspirin prophylaxis during pregnancy, gestational diabetes mellitus, any breastfeeding and the interaction between clinical visits and study group.

CHT: chronic hypertension; EMM: estimated marginal means; HDP: hypertensive disorder of pregnancy;

*: significant difference between 6-12 weeks and 6 months;

#: significant difference between 6-12 weeks and 12 months

†: significant difference between 6 months and 12 months

**Table S6 Sensitivity analysis of estimated marginal means and effect size of cardiac hemodynamics and geometry between women at high risk of pre-eclampsia with and without hypertensive disorder of pregnancy (HDP) at each study visit, stratified by exclusion criteria**

| **Visit** | **Exclusion of participants with newly-onset CHT** | | | | | **Exclusion of participants with** **previous history of HDP** | | | | | **Exclusion of participants with newly-onset CHT and previous history of HDP** | | | | |
| --- | --- | --- | --- | --- | --- | --- | --- | --- | --- | --- | --- | --- | --- | --- | --- |
|  | **Non-HDP** | **HDP** | **EMM (95%CI)** | **Cohen's d (95% CI)** | **P** | **Non-HDP** | **HDP** | **EMM (95%CI)** | **Cohen's d**  **(95% CI)** | **P** | **Non-HDP** | **HDP** | **EMM (95%CI)** | **Cohen's d (95%)** | **P** |
| **Hemodynamics** | | | | | | | | | | | | | | | |
| **MAP (mmHg)** | | | | | | | | | | | | | | | |
| **6-12 weeks** | 85.5 (79.0 to 92.0) | 93.4 (86.7 to 100.1) | 7.9 (4.8 to 10.9) | 1.69 (1.03 to 2.34) | **<0.001** | 83.1 (76.2 to 90.0) | 90.2 (83.0 to 97.5) | 7.1 (3.7 to 10.6) | 1.56 (0.82 to 2.31) | **<0.001** | 83.0 (76.7 to 89.3) | 89.8 (83.1 to 96.5) | 6.8 (3.5 to 10.1) | 1.49 (0.77 to 2.21) | **<0.001** |
| **6 months** | 85.4 (78.8 to 91.9) | 92.6 (85.9 to 99.3) | 7.2 (4.1 to 10.3) | 1.54 (0.89 to 2.20) | **<0.001** | 83.2 (76.3 to 90.1) | 90.0 (82.7 to 97.2) | 6.7 (3.3 to 10.1) | 1.47 (0.72 to 2.21) | **<0.001** | 83.1 (76.8 to 89.4) | 89.0 (82.3 to 95.7) | 5.9 (2.6 to 9.2) | 1.29 (0.56 to 2.01) | **<0.001** |
| **12 months** | 85.9 (79.4 to 92.4) | 92.6 (85.8 to 99.3) | 6.7 (3.5 to 9.8) | 1.43 (0.75 to 2.11) | **<0.001** | 83.5 (76.6 to 90.4) | 90.8 (83.6 to 98.1) | 7.3 (3.9 to 10.8) | 1.60 (0.84 to 2.37) | **<0.001** | 83.3 (77.0 to 89.6) | 89.9 (83.2 to 96.6) | 6.6 (3.2 to 10.0) | 1.45 (0.71 to 2.19) | **<0.001** |
| **P (between visits)** | 0.662 | 0.699 |  |  |  | 0.794 | 0.735 |  |  |  | 0.896 | 0.723 |  |  |  |
| **SVR (dynes⋅sec/cm⁵)** | | | | | | | | | | | | | | | |
| **6-12 weeks** | 1784.45 (1552.27 to 2016.64) | 1939.45 (1695.58 to 2183.33) | 155.00 (26.85 to 283.15) | 0.57 (0.10 to 1.05) | **0.019** | 1703.23 (1479.71 to 1926.76) | 1842.60 (1600.91 to 2084.30) | 139.37 (5.72 to 273.02) | 0.50 (0.02 to 0.98) | **0.043** | 1692.41 (1468.56 to 1916.25) | 1831.28 (1587.20 to 2075.37) | 138.87 (1.58 to 276.17) | 0.52 (0.01 to 1.03) | 0.049 |
| **6 months** | 1748.63 (1516.43 to 1980.83) | 1910.86 (1667.56 to 2154.17) | 162.23 (35.21 to 289.25) | 0.60 (0.13 to 1.07) | **0.013** | 1673.60 (1450.16 to 1897.04) | 1779.81 (1538.78 to 2020.84) | 106.21 (-26.23 to 238.65) | 0.38 (-0.09 to 0.86) | 0.118 | 1664.29 (1440.52 to 1888.07) | 1775.93 (1532.64 to 2019.22) | 111.63 (-24.20 to 247.47) | 0.42 (-0.09 to 0.93) | 0.11 |
| **12 months** | 1781.27 (1548.36 to 2014.19) | 1875.19 (1628.30 to 2122.07) | 93.91 (-39.97 to 227.80) | 0.35 (-0.15 to 0.84) | 0.171 | 1703.02 (1478.46 to 1927.57) | 1850.36 (1607.77 to 2092.94) | 147.34 (10.07 to 284.61) | 0.53 (0.04 to 1.03) | **0.037** | 1703.04 (1478.20 to 1927.88) | 1798.22 (1553.21 to 2043.23) | 95.18 (-45.76 to 236.11) | 0.36 (-0.17 to 0.88) | 0.188 |
| **P (between visits)** | 0.528 | 0.64 |  |  |  | 0.651 | 0.534 |  |  |  | 0.546 | 0.728 |  |  |  |
| **Cardiac geometry** | | | | | | | | | | | | | | | |
| **LVMI (g/m^2^)** | | | | | | | | | | | | | | | |
| **6-12 weeks** | 67.46 (59.56 to 75.37) | 71.98 (63.75 to 80.22) | 4.52 (0.47 to 8.57) | 0.58 (0.06 to 1.11) | **0.03** | 70.30 (63.63 to 76.98) | 75.74 (68.55 to 82.93) | 5.44 (1.54 to 9.33) | 0.68 (0.19 to 1.16) | **0.007** | 68.95 (61.59 to 76.30) | 73.98 (66.03 to 81.92) | 5.03 (0.77 to 9.29) | 0.65 (0.10 to 1.19) | **0.022** |
| **6 months** | 64.13 (56.23 to 72.03) | 68.77 (60.53 to 77.01) | 4.64 (0.59 to 8.69) | 0.60 (0.08 to 1.12) | **0.026** | 66.76 (60.08 to 73.44) | 72.10 (64.91 to 79.29) | 5.34 (1.43 to 9.25) | 0.67 (0.18 to 1.15) | **0.008** | 65.32 (57.96 to 72.68) | 70.68 (62.74 to 78.63) | 5.36 (1.11 to 9.62) | 0.69 (0.14 to 1.24) | **0.015** |
| **12 months** | 62.97 (55.06 to 70.87) | 67.46 (59.22 to 75.69) | 4.49 (0.44 to 8.54) | 0.58 (0.06 to 1.10) | **0.031** | 66.17 (59.48 to 72.86) | 71.02 (63.78 to 78.25) | 4.85 (0.83 to 8.87) | 0.60 (0.10 to 1.11) | **0.019** | 64.34 (56.98 to 71.69) | 69.56 (61.62 to 77.51) | 5.23 (0.97 to 9.49) | 0.67 (0.12 to 1.22) | **0.017** |
| **P (between visits)** | <0.001 *# | 0.043 # |  |  |  | <0.001 *# | 0.0485 |  |  |  | <0.001 *# | 0.0756 |  |  |  |
| **RWT** | | | | | | | | | | | | | | | |
| **6-12 weeks** | 0.28 (0.25 to 0.32) | 0.32 (0.28 to 0.36) | 0.04 (0.01 to 0.06) | 0.68 (0.26 to 1.11) | **0.002** | 0.29 (0.25 to 0.33) | 0.32 (0.28 to 0.36) | 0.03 (0.01 to 0.05) | 0.54 (0.10 to 0.97) | **0.017** | 0.29 (0.25 to 0.32) | 0.32 (0.28 to 0.36) | 0.03 (0.01 to 0.06) | 0.64 (0.18 to 1.09) | **0.007** |
| **6 months** | 0.28 (0.24 to 0.32) | 0.30 (0.26 to 0.34) | 0.02 (-0.00 to 0.04) | 0.37 (-0.06 to 0.79) | 0.093 | 0.28 (0.25 to 0.32) | 0.30 (0.26 to 0.34) | 0.02 (-0.01 to 0.04) | 0.31 (-0.13 to 0.75) | 0.166 | 0.28 (0.25 to 0.32) | 0.30 (0.26 to 0.34) | 0.02 (-0.01 to 0.04) | 0.33 (-0.12 to 0.79) | 0.154 |
| **12 months** | 0.27 (0.23 to 0.31) | 0.30 (0.26 to 0.35) | 0.03 (0.01 to 0.06) | 0.59 (0.15 to 1.04) | **0.01** | 0.27 (0.24 to 0.31) | 0.30 (0.26 to 0.34) | 0.03 (0.01 to 0.05) | 0.56 (0.11 to 1.01) | **0.016** | 0.27 (0.23 to 0.31) | 0.31 (0.26 to 0.35) | 0.04 (0.01 to 0.06) | 0.67 (0.19 to 1.14) | **0.007** |
| **P (between visits)** | 0.153 | 0.233 |  |  |  | 0.078 | 0.4 |  |  |  | 0.0492 | 0.3145 |  |  |  |

Adjusted by clinical visits (study timepoints), study group, age, height, weight, smoking, family history of CVD, parity, history of previous HDP, aspirin prophylaxis during pregnancy, gestational diabetes mellitus, any breastfeeding and the interaction between clinical visits and study group.

MAP: mean arterial pressure; SVR: systemic vascular resistance; RWT: relative wall thickness; CHT: chronic hypertension; EMM: estimated marginal means; HDP: hypertensive disorder of pregnancy;

*: significant difference between 6-12 weeks and 6 months;

#: significant difference between 6-12 weeks and 12 months

†: significant difference between 6 months and 12 months

**Table S****7 Sensitivity analysis of estimated marginal means and effect size of cardiac systolic and diastolic function between women at high risk of pre-eclampsia with and without hypertensive disorder of pregnancy (HDP) at each study visit, stratified by exclusion criteria.**

|  | **Exclusion of participants with newly-onset CHT** | | | | | | **Exclusion of participants with previous history of HDP** | | | | | | **Exclusion of participants with newly-onset CHT and previous history of HDP** | | | | | |
| --- | --- | --- | --- | --- | --- | --- | --- | --- | --- | --- | --- | --- | --- | --- | --- | --- | --- | --- |
| **Visit** | **Non-HDP** | **HDP** | **EMM (95%CI)** | **Cohen's d (95%)** | **P-value** | **Non-HDP** | | **HDP** | **EMM (95%CI)** | **Cohen's d (95%)** | **P-value** | **Non-HDP** | | **HDP** | **EMM (95%CI)** | **Cohen's d (95%)** | **P-value** |  |
| **Systolic function** | | | | | | | | | | | | | | | | | | |
| **LVEF** | | | | | | | | | | | | | | | | | | |
| **6-12 weeks** | 0.58 (0.55 to 0.61) | 0.56 (0.53 to 0.59) | -0.02 (-0.04 to 0.00) | -0.40 (-0.82 to 0.02) | 0.064 | 0.58 (0.56 to 0.61) | | 0.56 (0.53 to 0.60) | -0.02 (-0.04 to -0.00) | -0.51 (-0.95 to -0.06) | 0.027 | 0.58 (0.56 to 0.61) | | 0.57 (0.53 to 0.60) | -0.02 (-0.04 to 0.00) | -0.41 (-0.87 to 0.04) | 0.078 |  |
| **6 months** | 0.59 (0.56 to 0.62) | 0.58 (0.55 to 0.61) | -0.01 (-0.03 to 0.00) | -0.32 (-0.74 to 0.09) | 0.132 | 0.60 (0.57 to 0.63) | | 0.58 (0.55 to 0.62) | -0.02 (-0.03 to 0.00) | -0.36 (-0.80 to 0.08) | 0.11 | 0.60 (0.57 to 0.62) | | 0.59 (0.56 to 0.62) | -0.01 (-0.03 to 0.01) | -0.26 (-0.71 to 0.19) | 0.264 |  |
| **12 months** | 0.60 (0.57 to 0.63) | 0.60 (0.56 to 0.63) | -0.01 (-0.03 to 0.01) | -0.21 (-0.64 to 0.23) | 0.357 | 0.61 (0.58 to 0.64) | | 0.60 (0.57 to 0.63) | -0.01 (-0.03 to 0.01) | -0.24 (-0.69 to 0.21) | 0.303 | 0.61 (0.58 to 0.64) | | 0.60 (0.57 to 0.64) | -0.00 (-0.02 to 0.02) | -0.10 (-0.56 to 0.36) | 0.676 |  |
| **P value** | <0.001 *# | 0.0170 # |  |  |  | <0.001 *#† | | 0.003 # |  |  |  | <0.001 *# | | 0.004 # |  |  |  |  |
| **MAPSE (cm)** | | | | | | | | | | | | | | | | | | |
| **6-12 weeks** | 1.54 (1.41 to 1.66) | 1.39 (1.26 to 1.52) | -0.14 (-0.21 to -0.08) | -0.94 (-1.39 to -0.49) | **<0.001** | 1.55 (1.44 to 1.67) | | 1.38 (1.25 to 1.50) | -0.18 (-0.25 to -0.11) | -1.17 (-1.63 to -0.71) | **<0.001** | 1.56 (1.44 to 1.67) | | 1.40 (1.27 to 1.52) | -0.16 (-0.23 to -0.09) | -1.05 (-1.54 to -0.56) | **<0.001** |  |
| **6 months** | 1.55 (1.43 to 1.67) | 1.43 (1.30 to 1.55) | -0.12 (-0.19 to -0.05) | -0.79 (-1.24 to -0.34) | **<0.001** | 1.55 (1.44 to 1.67) | | 1.43 (1.31 to 1.56) | -0.12 (-0.19 to -0.05) | -0.79 (-1.25 to -0.33) | **<0.001** | 1.56 (1.44 to 1.67) | | 1.43 (1.30 to 1.55) | -0.13 (-0.20 to -0.06) | -0.85 (-1.33 to -0.36) | **<0.001** |  |
| **12 months** | 1.52 (1.40 to 1.65) | 1.46 (1.33 to 1.59) | -0.06 (-0.13 to 0.01) | -0.39 (-0.87 to 0.08) | 0.106 | 1.53 (1.42 to 1.65) | | 1.46 (1.33 to 1.59) | -0.07 (-0.15 to -0.00) | -0.50 (-0.98 to -0.01) | **0.046** | 1.54 (1.42 to 1.65) | | 1.48 (1.35 to 1.61) | -0.06 (-0.13 to 0.02) | -0.37 (-0.87 to 0.14) | 0.156 |  |
| **P value** | 0.524 | 0.183 |  |  |  | 0.621 | | 0.086 |  |  |  | 0.541 | | 0.121 |  |  |  |  |
| **Global longitudinal strain** | | | | | | | | | | | | | | | | | | |
| **6-12 weeks** | -0.25 (-0.26 to -0.24) | -0.24 (-0.25 to -0.22) | 0.01 (0.01 to 0.02) | 0.81 (0.34 to 1.27) | **<0.001** | -0.25 (-0.26 to -0.23) | | -0.23 (-0.25 to -0.22) | 0.01 (0.01 to 0.02) | 0.86 (0.37 to 1.34) | **<0.001** | -0.25 (-0.26 to -0.23) | | -0.23 (-0.25 to -0.22) | 0.01 (0.00 to 0.02) | 0.82 (0.32 to 1.32) | **0.002** |  |
| **6 months** | -0.26 (-0.27 to -0.24) | -0.24 (-0.25 to -0.23) | 0.02 (0.01 to 0.02) | 1.02 (0.55 to 1.49) | **<0.001** | -0.26 (-0.27 to -0.24) | | -0.24 (-0.26 to -0.23) | 0.01 (0.01 to 0.02) | 0.87 (0.38 to 1.35) | **<0.001** | -0.25 (-0.27 to -0.24) | | -0.24 (-0.25 to -0.23) | 0.01 (0.01 to 0.02) | 0.91 (0.41 to 1.42) | **<0.001** |  |
| **12 months** | -0.26 (-0.27 to -0.24) | -0.25 (-0.26 to -0.24) | 0.01 (-0.00 to 0.01) | 0.39 (-0.11 to 0.89) | 0.129 | -0.25 (-0.27 to -0.24) | | -0.25 (-0.26 to -0.23) | 0.01 (-0.00 to 0.02) | 0.45 (-0.06 to 0.96) | 0.084 | -0.25 (-0.27 to -0.24) | | -0.25 (-0.26 to -0.24) | 0.01 (-0.00 to 0.01) | 0.36 (-0.17 to 0.89) | 0.183 |  |
| **P value** | <0.001 *# | 0.001 #† |  |  |  | <0.001 *# | | 0.001 # |  |  |  | <0.001 *# | | 0.001 # |  |  |  |  |
| **Diastolic function** | | | | | | | | | | | | | | | | | | |
| **Mitral valve E/A** | | | | | | | | | | | | | | | | | | |
| **6-12 weeks** | 1.39 (1.12 to 1.66) | 1.26 (0.99 to 1.54) | -0.13 (-0.25 to 0.00) | -0.60 (-1.20 to 0.00) | 0.053 | 1.39 (1.14 to 1.63) | | 1.21 (0.95 to 1.47) | -0.18 (-0.30 to -0.05) | -0.84 (-1.44 to -0.24) | **0.007** | 1.39 (1.15 to 1.64) | | 1.24 (0.98 to 1.50) | -0.15 (-0.29 to -0.02) | -0.72 (-1.34 to -0.10) | **0.024** |  |
| **6 months** | 1.45 (1.18 to 1.71) | 1.36 (1.08 to 1.63) | -0.09 (-0.22 to 0.04) | -0.43 (-1.05 to 0.19) | 0.177 | 1.44 (1.20 to 1.69) | | 1.31 (1.05 to 1.57) | -0.14 (-0.27 to -0.00) | -0.65 (-1.27 to -0.02) | **0.044** | 1.45 (1.21 to 1.69) | | 1.34 (1.08 to 1.60) | -0.11 (-0.24 to 0.03) | -0.51 (-1.15 to 0.12) | 0.116 |  |
| **12 months** | 1.49 (1.23 to 1.76) | 1.32 (1.04 to 1.60) | -0.17 (-0.32 to -0.03) | -0.82 (-1.51 to -0.13) | **0.022** | 1.50 (1.25 to 1.75) | | 1.29 (1.02 to 1.56) | -0.21 (-0.36 to -0.06) | -1.01 (-1.71 to -0.30) | **0.006** | 1.51 (1.26 to 1.75) | | 1.31 (1.04 to 1.58) | -0.20 (-0.35 to -0.05) | -0.93 (-1.64 to -0.22) | **0.012** |  |
| **P value** | 0.004 # | 0.242 |  |  |  | 0.002 # | | 0.208 |  |  |  | 0.004 # | | 0.222 |  |  |  |  |
| **LAEF** | | | | | | | | | | | | | | | | | | |
| **6-12 weeks** | 0.41 (0.38 to 0.45) | 0.39 (0.35 to 0.43) | -0.02 (-0.05 to -0.00) | -0.40 (-0.79 to -0.01) | **0.045** | 0.43 (0.40 to 0.46) | | 0.40 (0.36 to 0.43) | -0.03 (-0.05 to -0.01) | -0.51 (-0.90 to -0.11) | **0.013** | 0.42 (0.39 to 0.45) | | 0.40 (0.37 to 0.44) | -0.02 (-0.05 to -0.00) | -0.42 (-0.83 to -0.00) | **0.05** |  |
| **6 months** | 0.43 (0.40 to 0.46) | 0.41 (0.37 to 0.44) | -0.02 (-0.05 to -0.00) | -0.41 (-0.80 to -0.02) | 0.043 | 0.44 (0.41 to 0.47) | | 0.42 (0.38 to 0.45) | -0.03 (-0.05 to -0.00) | -0.46 (-0.85 to -0.06) | **0.026** | 0.44 (0.41 to 0.47) | | 0.42 (0.39 to 0.45) | -0.02 (-0.04 to 0.00) | -0.35 (-0.76 to 0.07) | 0.103 |  |
| **12 months** | 0.48 (0.45 to 0.51) | 0.43 (0.40 to 0.47) | -0.05 (-0.07 to -0.02) | -0.79 (-1.21 to -0.38) | **<0.001** | 0.49 (0.46 to 0.52) | | 0.45 (0.41 to 0.48) | -0.04 (-0.07 to -0.02) | -0.74 (-1.15 to -0.32) | **<0.001** | 0.49 (0.46 to 0.52) | | 0.44 (0.41 to 0.48) | -0.05 (-0.07 to -0.02) | -0.81 (-1.24 to -0.38) | **<0.001** |  |
| **P value** | <0.001 *#† | 0.01 # |  |  |  | <0.001 *#† | | 0.01 #† |  |  |  | <0.001 *#† | | 0.01 # |  |  |  |  |
| **LAEI** | | | | | | | | | | | | | | | | | | |
| **6-12 weeks** | 0.74 (0.63 to 0.85) | 0.68 (0.56 to 0.80) | -0.06 (-0.14 to 0.02) | -0.29 (-0.68 to 0.09) | 0.138 | 0.78 (0.67 to 0.88) | | 0.69 (0.57 to 0.81) | -0.08 (-0.16 to -0.00) | -0.42 (-0.81 to -0.02) | **0.041** | 0.77 (0.66 to 0.88) | | 0.70 (0.58 to 0.83) | -0.07 (-0.15 to 0.02) | -0.33 (-0.75 to 0.08) | 0.117 |  |
| **6 months** | 0.78 (0.67 to 0.90) | 0.73 (0.61 to 0.85) | -0.05 (-0.13 to 0.02) | -0.27 (-0.65 to 0.12) | 0.178 | 0.82 (0.71 to 0.93) | | 0.75 (0.63 to 0.87) | -0.07 (-0.15 to 0.01) | -0.33 (-0.73 to 0.06) | 0.1 | 0.81 (0.71 to 0.92) | | 0.77 (0.64 to 0.89) | -0.05 (-0.13 to 0.03) | -0.24 (-0.66 to 0.17) | 0.251 |  |
| **12 months** | 0.97 (0.86 to 1.08) | 0.82 (0.69 to 0.94) | -0.15 (-0.23 to -0.07) | -0.75 (-1.16 to -0.34) | **<0.001** | 1.00 (0.90 to 1.11) | | 0.87 (0.75 to 0.99) | -0.14 (-0.22 to -0.05) | -0.69 (-1.10 to -0.27) | **0.001** | 1.00 (0.89 to 1.11) | | 0.85 (0.73 to 0.97) | -0.15 (-0.24 to -0.06) | -0.76 (-1.19 to -0.33) | **<0.001** |  |
| **P value** | <0.001 #† | 0.02 # |  |  |  | <0.001 #† | | 0.02 # |  |  |  | <0.001 #† | | 0.02 # |  |  |  |  |

Adjusted by clinical visits (study timepoints), study group, age, height, weight, smoking, family history of CVD, parity, history of previous HDP, aspirin prophylaxis during pregnancy, gestational diabetes mellitus, any breastfeeding and the interaction between clinical visits and study group.

CHT: chronic hypertension; EMM: estimated marginal means; HDP: hypertensive disorder of pregnancy; MAPSE: mitral annular plane systolic excursion; LVEF: left ventricular ejection fraction; LAEF: left atrial emptying fraction; LAEI: left atrial expansion index;

*: significant difference between 6-12 weeks and 6 months;

#: significant difference between 6-12 weeks and 12 months

†: significant difference between 6 months and 12 months

**Table S8 Estimated marginal means of cardiovascular profile for women with different types of hypertensive disorder of pregnancy.**

| **Visit** | **HR-non-HDP** | **Gestational hypertension** | **Late-onset PE** | **Early-onset PE** | **P-value for group comparison at each visit** |
| --- | --- | --- | --- | --- | --- |
| **Mean arterial pressure (mmHg)** |  |  |  |  |  |
| 6-12 weeks | 85.16 (79.82-90.5) | 95.06 (89.04-101.08) | 92.36 (86.45-98.28) | 91.34 (84.78-97.91) | <0.001 ab |
| 6 months | 84.97 (79.55-90.39) | 95.09 (88.63-101.55) | 93.03 (86.42-99.64) | 89.54 (82.21-96.88) | <0.001 ab |
| 12 months | 85.55 (79.99-91.11) | 94.96 (87.76-102.16) | 93.57 (85.66-101.49) | 90.67 (82.05-99.28) | <0.001 ab |
| P value for clinical visits comparison | 0.54 | 0.98 | 0.91 | 0.7 |  |
| **Systemic vascular resistance (dynes⋅sec/cm⁵)** |  |  |  |  |  |
| 6-12 weeks | 1752.17 (1540.53-1963.81) | 1874.28 (1626.67-2121.9) | 2033.56 (1782.62-2284.5) | 1809.43 (1524.99-2093.88) | 0.04 |
| 6 months | 1715.71 (1502.65-1928.77) | 1837.58 (1581.91-2093.25) | 1946.26 (1682.97-2209.55) | 1874.37 (1582.55-2166.19) | 0.05 |
| 12 months | 1738.84 (1522.3-1955.37) | 1881.99 (1610.51-2153.47) | 2061.14 (1763.25-2359.03) | 1741.84 (1419.58-2064.1) | 0.02 b |
| P value for clinical visits comparison | 0.58 | 0.88 | 0.63 | 0.61 |  |
| **Left ventricular mass index (g/m^2^)** |  |  |  |  |  |
| 6-12 weeks | 70.65 (64.34-76.97) | 77.21 (69.84-84.59) | 74.97 (67.52-82.43) | 72.97 (64.71-81.23) | 0.046 |
| 6 months | 67.38 (61.01-73.75) | 74.66 (67.02-82.29) | 69.88 (62.03-77.74) | 67.63 (58.93-76.34) | 0.042 a |
| 12 months | 66.66 (60.2-73.12) | 74.33 (66.2-82.46) | 67.42 (58.5-76.34) | 69.05 (59.4-78.7) | 0.037 a |
| P value for clinical visits comparison | <0.001 *# | 0.48 | 0.07 | 0.31 |  |
| **Global longitudinal strain** |  |  |  |  |  |
| 6-12 weeks | -0.25 (-0.26 to -0.24) | -0.24 (-0.25 to -0.23) | -0.23 (-0.24 to -0.22) | -0.24 (-0.25 to -0.22) | 0.002 b |
| 6 months | -0.26 (-0.27 to -0.25) | -0.25 (-0.26 to -0.23) | -0.24 (-0.25 to -0.22) | -0.25 (-0.26 to -0.23) | <0.001 b |
| 12 months | -0.26 (-0.27 to -0.25) | -0.24 (-0.26 to -0.23) | -0.25 (-0.27 to -0.23) | -0.26 (-0.28 to -0.24) | 0.038 a |
| P value for clinical visits comparison | <0.001 *# | 0.64 | 0.02 # | 0.017 # |  |

Adjusted by clinical visits (study timepoints), study group, age, height, weight, smoking, family history of CVD, parity, history of previous HDP, aspirin prophylaxis during pregnancy, gestational diabetes mellitus, any breastfeeding and the interaction between clinical visits and study group.

HR-non-HDP: high-risk women of preeclampsia who did not develop hypertensive disorder of pregnancy; HDP: hypertensive disorder of pregnancy;

PE: Preeclampsia

significant difference: * visit 1 vs visit 2; # visit 1 vs visit 3; † visit 2 vs visit 3

significant difference: a: HR-non-HDP vs GHT; b HR-non-HDP vs Late-onset PE; c HR-non-HDP vs Early-onset PE; d GHT vs Late-onset PE; e GHT vs Early-onset PE; f: Early-onset PE vs Early-onset PE;

**Reference**

1.	Wang X, Sahota DS, Wong L, Nguyen-Hoang L, Chen Y, Tai AST, Liu F, Lau SL, Lee APW and Poon LC. Prediction of pre-eclampsia using maternal hemodynamic parameters at 12 + 0 to 15 + 6 weeks. *Ultrasound Obstet Gynecol*. 2025;65:173-182.

2.	Lang RM, Badano LP, Mor-Avi V, Afilalo J, Armstrong A, Ernande L, Flachskampf FA, Foster E, Goldstein SA, Kuznetsova T, Lancellotti P, Muraru D, Picard MH, Rietzschel ER, Rudski L, Spencer KT, Tsang W and Voigt JU. Recommendations for cardiac chamber quantification by echocardiography in adults: an update from the American Society of Echocardiography and the European Association of Cardiovascular Imaging. *Eur Heart J Cardiovasc Imaging*. 2015;16:233-70.

3.	Hedberg P, Selmeryd J, Leppert J and Henriksen E. Long-term prognostic impact of left atrial volumes and emptying fraction in a community-based cohort. *Heart*. 2017;103:687-693.

**Figure S1**

Trajectory of mean arterial pressure (a) and mean left ventricular mass index (b) with 95%CI among four outcome groups in the postpartum (PN) period, including high risk without hypertensive disorder of pregnancy (HDP), gestational hypertension (GH), late-onset pre-eclampsia (PE) and early-onset PE.
